# High-Quality, High-Impact Augmented Virtuality System for the Evaluation of the Influence of Context on Consumer Perception and Hedonics: A Case Study in a Sports Bar Environment

**DOI:** 10.3390/foods14223950

**Published:** 2025-11-18

**Authors:** João Pedro Marques, José Carlos Ribeiro, Rui Costa Lima, Luís Baião, Bruna Barbosa, Célia Rocha, Luís Miguel Cunha

**Affiliations:** 1 GreenUPorto—Sustainable Agrifood Production Research Centre/Inov4Agro, DGAOT, Faculty of Sciences, University of Porto, Campus de Vairão, Rua da Agrária 747, 4485-646 Vairão, Portugaljoribeiro@fc.up.pt (J.C.R.);; 2 Sense Test, Lda, Rua Zeferino Costa 341, 4400-345 Vila Nova de Gaia, Portugal; rcl@sensetest.pt; 3 Faculty of Nutrition and Food Sciences, University of Porto, Rua do Campo Alegre 823, 4150-180 Porto, Portugal

**Keywords:** contextual research, extended reality, food consumption environment, immersive, mixed reality, sensory science

## Abstract

The adoption of immersive technologies is increasing in sensory, consumer, and marketing research, yet existing extended reality (XR) systems face limitations in realism, ease of product interaction, presence, data collection, and scalability. This study presents Sense-AV, an augmented virtuality (AV) system designed for large-scale sensory and consumer tests with enhanced immersion and realism. 102 participants evaluated two foods and one beverage across two sessions: a conventional sensory booth and the Sense-AV system, which simulated a sports bar environment. Real-time data collection was supported through API-linked mobile questionnaires, audio prompts via the head-mounted display (HMD), and open comments recorded by voice. Sense-AV was rated highly for usability, efficiency, satisfaction, presence, and sensory awareness. Older participants reported greater ease in handling products, while some difficulties with mobile input were noted but had minimal impact on the overall user experience (UX). Interviews emphasized immersion, intuitive use, and minor technical adjustments. No significant differences in overall product liking were found between methods, except for the mayonnaise, which scored higher in the immersive setting. Although food intake was lower in Sense-AV, oral feedback was more detailed and expressive. The system demonstrates innovation by improving realism and external validity in large-scale sensory evaluations.

## 1. Introduction

Recent advances in technology have reshaped how businesses engage with consumers, as outlined by Kotler et al. [1] in Marketing 6.0: The Future is Immersive. This framework expands upon previous marketing models by integrating human-centred, digital, and immersive technologies that aim to create multi-sensory and personalised user experiences (UXs) [1]. Within this context, immersive technologies such as Virtual Reality (VR) and Mixed Reality (MR), which encompass both Augmented Reality (AR) and Augmented Virtuality (AV), have gained prominence as tools that deepen consumer engagement, improve product interaction, and enhance decision-making processes [1,2,3]. These technologies, collectively referred to as Extended Reality (XR), align with the Virtuality Continuum, first introduced by Milgram and Kishino [4]. VR offers an immersive experience by transporting users into either a computer-generated or real-world recorded environment, effectively and completely occluding the physical surroundings [3,5,6]. This is typically achieved through head-mounted displays (HMDs) that provide a visual and auditory immersion, often complemented by additional devices such as motion controllers or data gloves [6]. AR refers to the overlay of digital content (e.g., images, videos, virtual items) onto the physical environment, augmenting rather than replacing the real-world view [7]. This technology is increasingly being adopted through mobile applications and AR glasses, with significant use cases across various sectors (e.g., healthcare, retail, education and gaming) [8,9,10,11]. By contrast, AV, a comparatively less-explored area, integrates real-world elements into primarily virtual environments, enriching user interaction by embedding physical references (e.g., real objects or users’ hands) within digital settings, often using markers or chroma key techniques [3,4,12].

In sensory and consumer science, the integration of immersive technologies presents novel opportunities, particularly for studying product evaluation and consumer responses through the simulation of different consumption contexts or scenarios [13,14,15]. This enhances ecological validity by creating a sense of presence in the desired context, which is crucial when assessing desire, preference, liking, emotion, and food choice [13,16,17]. The lack of contextual information in conventional sensory evaluation methodologies can lead to inaccurate predictions of food-related behaviours [18] and has been identified as one of the main factors contributing to the inability of consumer methodologies to predict the success of new products on the market [19,20].

Numerous studies have explored the use of VR, AV, and AR in the fields of sensory science, consumer perception and behaviour. Most studies have applied VR (*n* = 25; 80.6%), while AV (*n* = 4; 12.9%) and AR (*n* = 2; 6.5%) have been less studied (Table S1). Specifically regarding AR, studies have investigated its potential to enrich product experiences by incorporating multisensory enhancements to shape consumer perception, sensory evaluation and behavioural responses [21,22]. In terms of VR and AV, studies have primarily focused on transforming the context environment to simulate a range of food scenarios, although they have also been used to explore consumer-related behaviours, such as food selection and purchasing decisions, as well as to investigate specific behavioural and psychological aspects (Table S1). Most studies rely on 3D content and real-time simulations (n = 18; 58.1%; Table S1), which may reduce presence by using unrealistic or game-like features [23,24]. While simple environments are easier to render with acceptable realism, complex or dynamic settings involving people, large crowds, or natural phenomena (e.g., wind or flowing water) remain difficult to replicate accurately, often resulting in lower presence, realism, and immersion [25,26]. Furthermore, with the application of VR, food is typically represented as 3D models rather than real items, and users often cannot see the actual food or their hands, which limits the realism of the experience (Table S1).

In terms of the data collection methodology, only a few studies have enabled participants to evaluate and respond directly within the virtual environment (usually by using handheld controllers or hand tracking), offering the advantage of a self-guided XR UX that avoids reliance on memory-based responses [27,28,29,30,31,32,33,34]. Many studies in this field overlook the assessment of UX and perceived presence, focusing instead on sensory evaluations and product–context congruence. Combined with typically small sample sizes and the need for participant guidance, these limitations restrict the ability to evaluate the realism or immersion of XR systems, raising concerns about their viability for large-scale sensory and consumer research.

Although still rarely used in this field, AV systems based on pre-recorded 360° videos of real environments appear to be a promising tool for sensory evaluations in different contexts, as they allow participants to interact with real food products in realistic settings while viewing recordings of real locations and actual people, which can potentially enhance contextual realism and ecological validity [27,29]. Despite their potential, this type of AV system often presents several limitations when not properly implemented, including visual artefacts (e.g., chroma key outlines), low video quality and inadequate filming specifications that lead to distortions in scale, height and depth perception, ultimately reducing realism [35,36]. Moreover, when participants are unable to complete sensory questionnaires without technical assistance, the need for intervention disrupts the experience, potentially compromising both immersion and the quality of the collected data [37].

In response to these challenges, the present study aimed to develop and validate the SenseVirtual augmented virtuality (Sense-AV) system, specifically designed to address the limitations commonly reported in sensory and consumer studies that utilise immersive technologies. The system was designed to offer a real-time first-person point of view (POV) in which users can see their real hands and real food products on a physical table, all integrated into a virtual environment (in this study, a sports bar), within a controlled yet ecologically valid setting.

## 2. Materials and Methods

### 2.1. System Setup and Architecture

**Figure 1:**
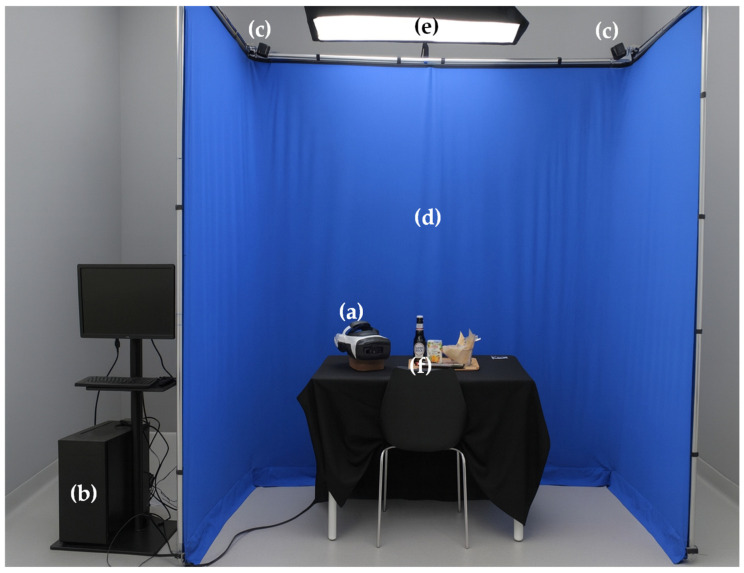
Overview of the Sense-AV system components and setup: (**a**) Varjo XR-4 focal edition HMD, (**b**) Workstation, (**c**) SteamVR Base Stations 2.0, (**d**) Chroma key structure, (**e**) Softbox-equipped LED light, (**f**) Participants’ sensory evaluation location.

As shown in Figure 1, the Sense-AV system comprises an integrated configuration of hardware, software, and environmental components designed to support immersive, ecologically valid sensory evaluations within context-specific virtual food consumption environments (e.g., a bar, restaurant, or public park). The system uses an HMD (Varjo, Helsinki, Finland) to present high-resolution 8 K (8192 × 4096 pixels), 360° videos of real-world scenarios, displayed within a blue chroma key area to ensure a high degree of realism. Concurrently, real food and beverage products are physically presented, consumed, and evaluated using user-friendly mobile questionnaires, while participants can see and interact with real-world elements (e.g., food, utensils, mobile device, hands) through the HMD, enabling seamless integration of the virtual and physical environments to ensure a natural interaction.

The Sense-AV system is structured into three main components: hardware configuration (Appendix A.1), software development, data capture, and synchronisation (Appendix A.2), and Environmental setup (Appendix A.3). Detailed descriptions of each component are provided in the appendices.

### 2.2. System Validation

#### 2.2.1. Participants

A total of 102 participants (60.8% female) aged between 18 and 65 years (mean age = 46.7 ± 12.6) were recruited via Sense Test’s consumer database, a sensory analysis and consumer testing company based at its central facility in Vila Nova de Gaia, Portugal. The participants were predominantly from the Porto metropolitan area in northern Portugal. Recruitment was based on participants’ willingness to engage in a sensory evaluation involving an AV system. Additionally, participants were required to be occasional or regular consumers of the products being evaluated. Participants were asked to refrain from eating, drinking, and smoking for at least one hour before participating in the sensory evaluation sessions. No prior familiarity with VR or MR technologies was required for inclusion. Familiarity with these technologies was assessed through a question administered before the evaluation began in the Sense-AV setting (Table S2).

All participants received a verbal briefing on the general aspects of the research before being asked to sign the informed consent form, which was approved by the Ethics Committee of the Faculty of Science at the University of Porto (no. CE2025/p45).

Sense Test ensures that participant recruitment was carried out through the company database, which guarantees the protection and confidentiality of data through a long-standing internal code of conduct. This code includes authorisation No. 2063/2009 from the National Data Protection Commission, subsequent adaptation to the General Data Protection Regulation (EU) 2016/679.

#### 2.2.2. Products

All tested products were commercially available items and were presented on a bamboo serving board (35 × 25 × 1.4 cm; width × height × depth), reinforcing contextual congruence with the sensory and behavioural expectations of a typical sports bar environment. The primary food item was a grilled sausage, served warm in a black rectangular tray. Each sausage (41.22 ± 1.84 g after grilling) was grilled for 4 min on each side on a pre-heated electric grill at 180 °C to ensure full cooking and consistent texture and flavour. A top-down plastic bottle of mayonnaise (with a nominal capacity of 450 mL), accompanied by a side portion of deep-fried chips (46.63 ± 14.37 g), presented in a bar-style stainless steel fry basket. Mayonnaise was served at room temperature, while chips were served immediately after frying (approximately 65 °C) to maintain their optimal texture.

For beverages, participants were given a choice between two options: Algarve orange nectar (200 mL, served in its original individual packaging with a straw at 6–8 °C) or a reduced-calorie light wheat beer (200 mL, served directly from the glass bottle at 6–8 °C).

Products and beverages were selected to reflect typical items and handling practices in a sports bar environment. Participants ate the sausage using a fork and knife, consumed the chips with their hands, drank the orange nectar through a straw, drank the beer directly from the bottle (opening it with a bottle opener), and handled the mayonnaise container. This experimental design enabled the testing of various food and beverage interaction modes while wearing the HMD, allowing for the assessment of feasibility and natural handling.

Both sessions were designed to resemble a casual consumption experience rather than a conventional sensory test. The order of food interaction was not strictly fixed, allowing participants to eat and drink in any order or to consume items simultaneously, a practice typically discouraged in conventional sensory analysis.

#### 2.2.3. Study Environments

**Figure 2:**
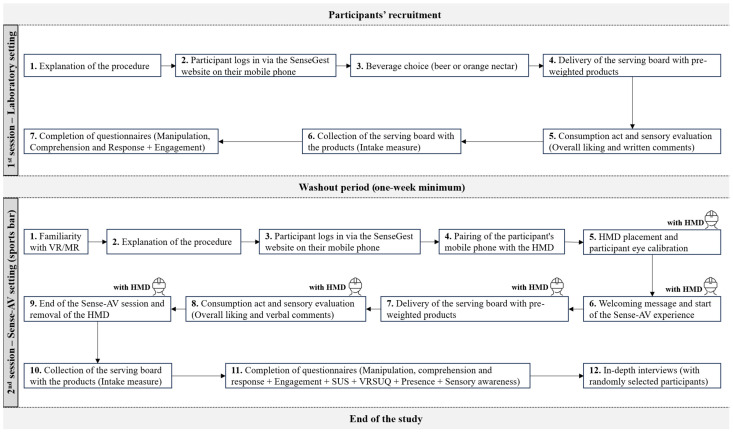
Study flowchart.

**Figure 3:**
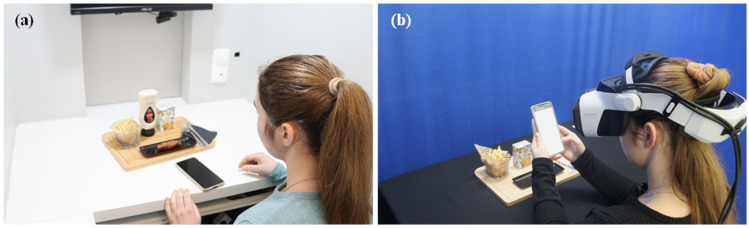
(**a**) A participant conducting the session in the laboratory setting (conventional sensory booth), (**b**) and in the Sense-AV setting.

Sensory evaluations were first conducted in a laboratory setting, followed by a session in the Sense-AV system (Figure 2). An interval of at least one week was established between sensory sessions to minimise carryover effects and reduce the influence of sensory memory on their assessments. Both sessions were conducted similarly, and product evaluations were completed on the participant’s mobile phone (Figure 3).

##### Laboratory Setting

The first sessions were conducted in individual sensory booths at Sense Test’s sensory evaluation laboratory, which is equipped in accordance with ISO 8589:2007 (Sensory analysis—General guidance for the design of test rooms) [38] and operates under a quality management system certified according to ISO 9001:2015 (Quality management systems—Requirements) [39]. Room temperature (19 ± 2 °C) was maintained throughout the sessions, and each booth was illuminated with uniform white lighting.

##### Sense-AV Setting

The second evaluation was conducted using the Sense-AV system, which featured a virtual simulation of a sports bar’s interior. The technical specifications of the recordings are detailed in Appendix A.2.

**Figure 4:**
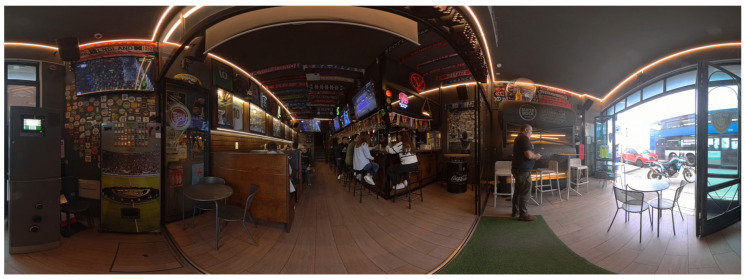
Virtual environment (sports bar) observed by participants during the Sense-AV session.

The virtual environment (Figure 4) featured a wooden bar counter with visible customers seated on the near side, regular empty tables and chairs, as the bar was not fully occupied, and booth-style seating with sofas, where people were also seated. During the Sense-AV experience, participants observed various scenes of social interaction, including customers conversing with one another and consuming different foods and beverages that were congruent with the specific setting. Multiple television screens were visible throughout the bar, each broadcasting football and tennis matches. Football-themed elements were present in the setting, including scarves representing various football clubs, football shirts, and a mural featuring the international football legend Diego Armando Maradona, all of which contributed to the authenticity of the environment. Participants could also view the bar entrance and the street scene outside by turning around. The background audio consisted of ambient music and overlapping conversations, which reinforced the atmosphere of a typical sports bar.

#### 2.2.4. Evaluation Methodologies

##### Sensory Evaluation

In the laboratory setting, the serving board with the food products was provided through the pass-through door by an assistant, while another assistant was positioned on the participant’s side of the booth to assist in case of any difficulties using their mobile devices or connecting to the internet. Each booth was sanitised after every session. After accessing the website and logging in, participants began the session. Participants were informed that they were free to consume the products in any order, eat as much as they wished, and select the order to assess them on their mobile device.

**Figure 5:**
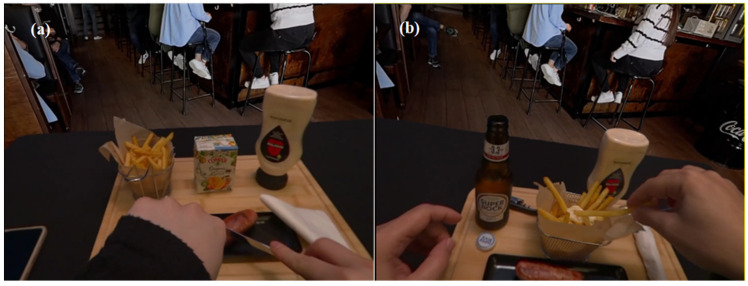
(**a**) A participant who chose orange nectar as their beverage, interacting with food items during the Sense-AV session, (**b**) A participant who chose beer as their beverage, interacting with food items during the Sense-AV session.

**Figure 6:**
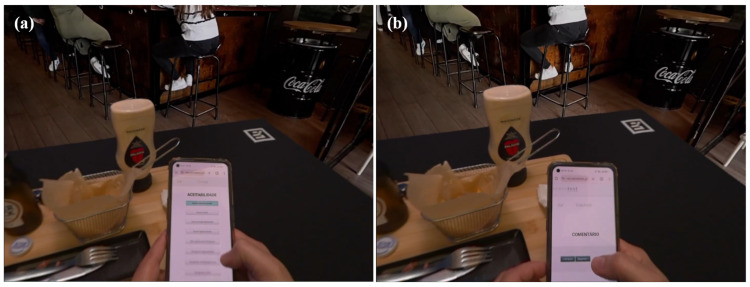
(**a**) A participant performing the hedonic evaluation, overall liking (*aceitabilidade*—in Portuguese), on their mobile phone during the Sense-AV session. (**b**) The same participant, while providing a verbal comment (*comentário*—in Portuguese) via audio recording during the Sense-AV session.

During sensory evaluations in the Sense-AV setting, the HMDs were cleaned, and the lenses were carefully wiped with screen-safe wipes after each use. The HMD was calibrated for each participant’s vision before the evaluation to ensure they could see and read the content displayed on the mobile screen. Before beginning the session, participants were asked if they could see the content clearly and whether the text was comprehensible on the mobile device. Adjustments were made, if necessary, to the distance between the participant’s face and the mobile device to ensure text visibility. Additionally, the screen brightness was optimised to provide adequate readability. Following a brief welcome screen with an audio greeting, the session started, and the virtual environment was presented against the chroma key background. From this point, participants engaged in product consumption (Figure 5) and conducted the sensory evaluation (Figure 6), with complete freedom to eat as much as they wanted and to choose the order of both consumption and assessment, similar to the laboratory session. A demonstration video of a participant consuming the products while using the Sense-AV system is provided in Video S1.

In both sessions, following the initial consumption, participants were asked to rate the overall liking of each product they tasted, except for the chips that served as an accompaniment to the mayonnaise, using a classic 9-point hedonic scale, ranging from 1 (“Dislike extremely”) to 9 (“Like extremely”) [40]. Participants were also asked to provide open comments. In the laboratory setting, these comments were written, as is typical in sensory evaluations. In contrast, in the Sense-AV setting, comments were provided verbally. This approach was adopted to facilitate a comparison of the speed and naturalness of participant feedback between the two evaluation formats. In the Sense-AV session, participants also received an audio prompt guiding them on how to proceed, in addition to the text displayed on the mobile device.

##### Food Intake

To assess differences in consumption between the laboratory and the Sense-AV session, the intake of all food products and the selected beverage were measured immediately following the session. Measurements for calculating the weight difference were taken using a HOTO QWCFC001 smart kitchen-calibrated weighing scale (Shanghai HOTO Technology Co., Ltd., Shanghai, China).

##### Post-Session Questionnaires

After each session, participants were asked to complete evaluation questionnaires.

In the laboratory session, participants were required to complete a specific questionnaire regarding the manipulation, comprehension, reading and response aspects (MCRRQ), where they rated the ease of the task on a 7-point scale, ranging from 1 (“Extremely difficult”) to 7 (“Extremely easy”) (Table S3). Subsequently, participants completed a 10-item Engagement Questionnaire (EQ) (from Hannum and Simons [41]), rated on a 7-point scale from “strongly disagree” to “strongly agree”. This questionnaire assessed three factors: “Active Involvement”, “Purposeful Intent”, and “Affective Value” (Table S4).

In the Sense-AV session, after removing the HMDs, participants were required to complete the MCRRQ and EQ, as in the laboratory session. Additionally, participants were asked to complete the System Usability Scale (SUS) questionnaire (from Brooke [42] and translated to Portuguese by Martins et al. [43]), as well as the “Efficiency” and “Satisfaction” subfactors from the Virtual Reality System Usability Questionnaire (VRSUQ) [44] (Table S5). Both were rated on a 7-point Likert scale ranging from 1 (“Strongly disagree”) to 7 (“Strongly agree”). SUS and VRSUQ scores were calculated and normalised to 0 to 100 scales. Participants also responded to a questionnaire on Presence and Sensory Awareness (PSAQ), developed based on the Multimodal Presence Scale (MPS) framework [45], assessing the subfactors: “Physical Presence,” “Social Presence,” and “Self-Presence,” as well as an extra subfactor entitled “Sensory Awareness” [46]. These were also rated on a 7-point Likert scale, ranging from 1 (“Strongly disagree”) to 7 (“Strongly agree”) (Table S6).

##### Semi-Structured Individual Interviews

Semi-structured individual interviews were conducted with randomly selected participants to gather additional insights into their perceptions and overall experience with the Sense-AV system. A total of 30 participants (16 women and 14 men; mean age, 44.7 ± 12.7 years), comprising 29.4% of the total participants, participated in the final interviews following the session in the Sense-AV system. The interview was divided into four sections: initial impressions, questions regarding immersion and presence, UX, and recommendations or suggestions for system improvement (Table S7). Each interview lasted between 5 and 15 min and was recorded using a Sony HDR-CX240E video camera (Sony Group Corporation, Tokyo, Japan). After being transcribed verbatim, the videos were deleted.

#### 2.2.5. Statistical Analysis

Statistical analyses were performed using SPSS software, version 29.0 (IBM, Armonk, New York, NY, USA). Data are reported as mean ± standard deviation (SD), as well as the percentage of positive responses (≥5 on 7-point scales).

The normality of the data was assessed using the Kolmogorov–Smirnov test, since the data were found not to be normally distributed, non-parametric tests were applied. The Wilcoxon signed-rank test was used for comparative analysis of overall liking, food intake, MCRRQ, and EQ scores between the laboratory session and the Sense-AV session.

The internal consistency of the SUS, the EQ, and the subscales of the VRSUQ and the PSAQ, were assessed using Cronbach’s α.

To assess the effects of social demographics and experience on MCRRQ, EQ, PSAQ and SUS and VRSUQ scores, participants were divided into different groups accordingly to sex (female; male), age (aged 49 or younger; aged between 50 and 65) and degree of experience with VR and MR (no previous experience; at least one engagement with one of these technologies).

The analysis of MCRRQ, EQ, PSAQ, SUS, and VRSUQ scores categorised by age group (younger adults, older adults), sex (male, female), and prior experience (no experience, experience) was conducted using the Mann–Whitney U test.

All analyses were performed with a 95% confidence level.

## 3. Results

### 3.1. Sensory Evaluation

#### 3.1.1. Overall Liking

As shown in Table 1, no significant differences were observed in overall liking scores between the sensory booth and the Sense-AV sessions for the grilled sausage (*p* = 0.869), nectar (*p* = 0.794), or beer (*p* = 0.850). However, a statistically significant difference was found for the mayonnaise, which received higher overall liking scores in the Sense-AV session (8.01 ± 0.88) compared to the sensory booth session (7.78 ± 1.19; *p* = 0.019).

#### 3.1.2. Open Comments

Across all four products, oral feedback obtained in the Sense-AV system setting was generally more elaborate, emotionally expressive, and lexically varied than the written comments collected in the sensory booth. On average, oral responses contained twice the number of words per participant and featured a greater use of evaluative qualifiers, sensory metaphors, contextual references, as well as more repetitions and intensifiers.

Written comments averaged 11.48 (±7.16) words for the sausage, while oral feedback reached 23.49 (±18.47) words. In both formats, frequently used descriptors included “tasty”, “pleasant”, and “juicy”. Repetition and emphasis (e.g., “I really like, really, really like”) appeared more frequently in oral comments. Oral responses in the Sense-AV setting also revealed greater lexical variety, including adjectives such as “spicy”, “nicely seasoned”, and “mild”, often accompanied by emotionally expressive remarks such as “almost addictive” or “good for a party”. These elaborations were usually less used in the written modality, which tended to prioritise clarity and conciseness. Additionally, critical observations concerning saltiness or greasiness appeared more frequently in the oral condition.

A similar pattern was observed for the mayonnaise. Written comments averaged 11.19 (±7.86) words, whereas oral feedback reached 22.43 (±17.09) words per participant. Common descriptors such as “creamy”, “tasty”, and “smooth” appeared in both modalities, although the oral condition prompted more nuanced and cautious expressions, including “a little acidic” and “not too strong”. Emphatic phrases and repetition (e.g., “really, really creamy and really, really tasty”) were again more frequent in the oral feedback. Temporal and situational references (e.g., “perfect for a summer barbecue”, “goes well with the chips”) were also more prevalent in the Sense-AV setting. Sensory metaphors and subjective impressions (e.g., “melts in the mouth”, “almost addictive”) also featured more prominently in the oral responses, while written comments tended to remain more objective and technical. Critical remarks concerning acidity or texture were more frequently voiced in the Sense-AV environment.

In the case of orange nectar, oral responses averaged 24.29 (±17.90) words, compared to 12.00 (±7.64) for written comments. Positive descriptors such as “tasty”, “fresh”, and “refreshing”, appeared frequently in both modalities. Repetitions and intensifiers (e.g., “really good”, “extremely tasty”) were more common in the oral responses. Metaphorical expressions and subjective impressions (e.g., “true orange taste”, “as if the orange had just been squeezed”, “it tasted like picking the orange straight from the tree”) were more frequent in the Sense-AV condition, whereas written comments remained more formulaic and predominantly descriptive. Temporal and contextual references (e.g., “goes well with what is being eaten”, “helps wash down the sausage”, “would be perfect for a healthy afternoon snack”) were also more prevalent in oral feedback. Critical remarks regarding sweetness or acidity were more frequent in the oral condition.

For beer, oral responses once more contained substantially more words (24.50 ± 14.51 vs. 13.77 ± 11.68) and were more expressive than written ones. As seen with the other products, spoken feedback featured greater lexical diversity and included more qualified and nuanced evaluations, such as “a bit weak” or “perhaps missing some flavour”. Positive descriptors such as “light”, “smooth”, and “refreshing” were frequent in both formats, as expected for a beer with lower calorie and alcohol content. Contextual references, such as consumption timing, setting, or food pairing (e.g., “ideal before lunch”, “ideal before work since it has a lower alcoholic content”, “enjoyed in a pleasant bar environment”, or “ideal when accompanied with the chips”), also appeared almost exclusively in the Sense-AV condition.

### 3.2. Food Intake

As shown in Table 2, significantly higher intake was observed in the sensory booth session for the grilled sausage (31.50 ± 9.94 g vs. 24.69 ± 11.85 g; *p* < 0.001), chips (23.24 ± 10.95 g vs. 19.86 ± 15.16 g; *p* = 0.023), nectar (151.02 ± 49.97 mL vs. 102.52 ± 53.65 mL; *p* < 0.001), and beer (144.12 ± 44.90 mL vs. 109.92 ± 47.04 mL; *p* < 0.001). However, no significant differences were found in mayonnaise consumption between the two sessions (*p* = 0.091).

### 3.3. Post-Session Questionnaires

#### 3.3.1. Manipulation, Comprehension, Reading and Response

Significantly lower scores were recorded in the Sense-AV session across all assessed subscales of the MCRRQ (Table 3), including manipulation (6.74 ± 0.42 vs. 5.97 ± 0.96; *p* < 0.001), reading through the phone (6.62 ± 0.63 vs. 4.70 ± 1.87; *p* < 0.001), responding through the phone (6.68 ± 0.61 vs. 5.38 ± 1.71; *p* < 0.001), understanding the information (6.86 ± 0.34 vs. 6.67 ± 0.63; *p* = 0.002), and providing the open comment (6.78 ± 0.50 vs. 6.26 ± 1.04; *p* < 0.001).

Positive response rates (scores ≥ 5) were analysed for both sessions across all subscales. In the sensory booth session, positive response rates were all above 98%. In the Sense-AV session, the positive response rates for manipulation, understanding the information, and providing open comments were all above 90%, while the subscales involving interaction with the mobile phone (reading and responding) were where participants encountered the greatest difficulties.

#### 3.3.2. Engagement

The internal consistency of the EQ was moderate in the laboratory session (Cronbach’s α = 0.612) and acceptable in the Sense-AV session (Cronbach’s α = 0.790). Regarding the EQ factor scores (Table 4), a significantly lower score was observed for Active Involvement in the Sense-AV session compared to the laboratory session (19.08 ± 2.77 vs. 19.57 ± 3.11; *p* = 0.024), while no significant differences were found between sessions for Purposeful Intent (*p* = 0.114) or Affective Value (*p* = 0.616).

#### 3.3.3. System Usability Scale (SUS) and Virtual Reality System Usability Questionnaire (VRSUQ)

The overall usability of the system, measured by the SUS (10 items), was 81.67 ± 14.41 (Cronbach’s α = 0.827).

For the VRSUQ, the Efficiency subscale (3 items) scored 84.36 ± 14.46 (Cronbach’s α = 0.577), and the Satisfaction subscale (3 items) scored 87.25 ± 15.86 (Cronbach’s α = 0.553).

Mean scores for individual items from SUS and VRSUQ subscales are presented in the Supplementary Material (Tables S8 and S9).

#### 3.3.4. Presence and Sensory Awareness

The Physical Presence subscale (5 items) had a mean score of 5.56 ± 1.46 (Cronbach’s α = 0.888), with 81.57% of responses classified as positive. The Social Presence subscale (5 items) yielded a mean of 5.06 ± 1.80 (Cronbach’s α = 0.806), with 68.62% positive responses. The Self-Presence subscale (5 items) had a mean of 5.27 ± 1.48 (Cronbach’s α = 0.922), with 74.31% of responses being positive. The Sensory Awareness component (4 items) exhibited a mean score of 5.52 ± 1.40 (Cronbach’s α = 0.783), with 78.92% positive responses.

Mean scores for individual items within the PSAQ components are presented in the Supplementary Material (Table S10).

#### 3.3.5. Effects of Age, Sex, and Experience on Questionnaire Responses

No significant effects were found for sex or prior VR/MR experience on any of the questionnaire measures. However, several age-related differences were observed (Table 5). Older participants (50–65 years) reported significantly higher scores than younger participants (18–49 years) in multiple aspects of the MCRRQ, including manipulation in both the booth (6.78 ± 0.40 vs. 6.70 ± 0.43; *p* = 0.042) and Sense-AV sessions (6.28 ± 0.69 vs. 5.67 ± 1.09; *p* = 0.005), as well as reading (5.16 ± 1.80 vs. 4.26 ± 1.86; *p* = 0.011), and responding (5.81 ± 1.56 vs. 4.98 ± 1.77; *p* = 0.003) through the phone in the Sense-AV session. In the EQ, older participants also scored significantly higher on Purposeful Intent during the booth session (26.95 ± 1.60 vs. 26.15 ± 2.31; *p* = 0.030). No other significant differences were observed between age groups across the remaining subscales of the MCRRQ, EQ, SUS, VRSUQ, or PSAQ.

### 3.4. Semi-Structured Individual Interviews

#### 3.4.1. Initial Impressions

Most participants (*n* = 26) described the experience as interesting, fun, engaging, or innovative. Several highlighted the novelty of the experience and expressed enthusiasm about repeating it. Many participants appreciated the realistic atmosphere of the bar, feeling that it added a social and familiar dimension to the sensory evaluation. A few older participants remarked that the setting evoked nostalgic memories, which enhanced the emotional impact of the experience, as one participant stated: “*It felt just like being in a real bar, like when I was younger. It was as if I actually felt younger again*” (P2, male, age 59).

However, a small number of participants (*n* = 4) described some initial confusion or strangeness upon entering the virtual environment, which typically subsided after a brief adaptation period, as one participant explained: “*At the beginning it feels a bit confusing. It takes a little while for the eyes to adjust to the system, about a minute or two*” (P11, male, age 41). A few participants (*n* = 3) also expressed that they enjoyed and found the overall experience exciting despite some discomfort related to the physical characteristics of the HMD, particularly its weight, as one noted: “*I found the experience amazing and felt fully integrated into the space. I even felt like people were looking at me as they passed by, which was interesting. The only issue was the weight of the glasses, as they kept slipping off because I started sweating*” (P30, female, age 55).

#### 3.4.2. Immersion and Presence

Most participants (*n* = 25) reported a strong sense of presence within the virtual bar, often describing the environment as realistic and coherent. For instance, one participant noted, “*The environment was pleasant, it was almost as if I were sitting at one of those tables with people around me. I felt completely at ease*” (P21, male, age 60), while another stated, “*I think it was a cosy place where people were chatting with each other or, in this case, eating, and I thought it was the perfect setting for the products that I had in front of me*” (P8, female, age 50). This sense of realism was reinforced by ambient details such as background sounds, the presence of other people, and typical bar elements. Participants frequently mentioned being aware of others in the scene, with some even attempting to interact or reporting a sensation of being observed. As one described, “*At a certain point, it felt like those people were real and existed, not just a recording. For example, when someone got up and walked past me towards the entrance, it felt like they were coming towards me*” (P18, male, age 26). Another participant noted, “*You could tell that people were talking to each other. I found it amusing when they threw bottles into the bin and you could hear the sound, as if the bottle were really being thrown away*” (P8, female, age 50).

However, a commonly reported limitation was the lack of interaction, which in some cases led to a perception of social isolation: “*It felt like I was there, and no one was aware of my presence*” (P12, female, age 40). Additionally, a few participants mentioned difficulty in visualising certain virtual elements: “*Some details, like the television, I couldn’t quite see. There was a football match on, but I couldn’t read the names or the scoreboard; that wasn’t very clear. But everything else, like the people, felt completely normal, as if it was real*” (P25, male, age 18).

A small number of participants (*n* = 3) also pointed out mismatches between the virtual and physical components, particularly regarding the position or size of the real table: “*I would only change the position of the table, to another place*” (P29, male, age 21), and “*The table where I was sitting seemed a bit too large for the space I was in*” (P10, female, age 47).

#### 3.4.3. User Experience

Participants generally managed the task well, with most reporting no significant problems when handling food products or using the mobile phone to evaluate the products. However, some technical and perceptual challenges were frequently reported. Visual clarity on the phone screen when viewed through the HMD emerged as a common issue (*n* = 11), with participants citing blurred text, particularly at the start of the experience: “*I had to find the right position to be able to read... But after that, it was easy*” (P30, female, age 55). Another participant noted: “*Perhaps holding the phone and realising that it is quite different from what we are used to might be a barrier to feeling fully immersed in the experience*” (P17, female, age 34). Similarly, difficulties adjusting focus were mentioned: “*If I moved the phone away, it became blurrier, if I brought it closer, things became a bit clearer and easier to understand*” (P18, male, age 26).

Some individuals also described altered depth perception or spatial disorientation when manipulating food or locating their mouths. Nevertheless, these issues were often resolved with brief periods of adaptation: “*The first time I picked up the cutlery, it felt a bit different, but as I relaxed and got into the environment, it was fine, completely fine*” (P29, female, age 41), and “*At first, it felt like I couldn’t quite find the opening (of the juice), but then it just took a few seconds to get used to it*” (P12, female, age 40).

Audio instructions were widely appreciated, with nearly all participants finding them clear and supportive. Preferences varied regarding the input method for open comments, though almost all the participants (*n* = 27) favoured audio input due to its more natural flow: “*It was just speaking. While that voice was asking for a comment, it felt just like I was sending a voice message, for example. It was just talking, and that was it. It was easy*” (P25, male, age 18). However, there were still some that said that they preferred written input (*n* = 3), as in the laboratory session, citing discomfort with speaking aloud or difficulty articulating specific thoughts: “*It was difficult. Writing is one thing, speaking is another. And I think I got confused… Putting thoughts together. When you’re writing, you think you need to mention this point, then that point, and you know you’re following a structure. But when you’re speaking… it feels like you get lost. And then there’s that awareness of… am I speaking too loudly? Can people hear me?*” (P15, female, age 43).

#### 3.4.4. Recommendations and Suggestions for Improvement

Nearly half of the participants did not suggest any improvements, indicating that the system was well implemented in its current form (*n* = 14). Among those who provided feedback, most suggestions focused on technical aspects rather than conceptual changes, often reinforcing issues they had previously identified as less satisfactory in earlier parts of the interview. The most mentioned issue concerned the clarity and focus of the HMD, particularly for reading content on the mobile phone screen (*n* = 6). Some participants also emphasised the need for a lighter or more comfortable HMD to improve overall physical comfort during use (*n* = 3). To enhance the sense of immersion, several participants recommended better alignment between physical and virtual elements, especially in relation to the table (*n* = 4), and more natural interactions with virtual characters present in the environment (*n* = 2). A few participants proposed alternatives to using the mobile phone for data entry, such as implementing voice commands, virtual buttons, or even paper-based questionnaires (*n* = 3). Finally, some highlighted the need to improve video resolution to make specific visual elements, such as television screens, clearer and more immersive (*n* = 2).

## 4. Discussion

### 4.1. Sensory Evaluation

#### 4.1.1. Overall Liking

In this study, overall liking scores for most of the tested food and beverage products did not differ significantly between the conventional sensory booth and the Sense-AV session, suggesting that the immersive environment did not affect hedonic perception. Nonetheless, mayonnaise received significantly higher liking scores in the Sense-AV session. This result aligns with previous findings indicating that immersive environments can enhance hedonic evaluations [46,47,48,49]. One possible explanation for this is that mayonnaise had the lowest overall liking score in the conventional sensory booth, which made the change in context more pronounced when compared to the higher scores of the other products. The strong congruence between the casual, social sports bar environment and the typical consumption context of mayonnaise, often used as a condiment with chips, likely contributed to this increased liking as well. The naturalistic setting and the physical manipulation of mayonnaise from a top-down bottle may have enhanced the realism of the experience, contributing to its increased appeal.

By contrast, the grilled sausage, orange nectar and beer showed no significant differences in liking between the two sessions. This finding is consistent with previous research suggesting that some products are evaluated consistently across different contexts [27,31,50,51]. Participants’ familiarity with sensory evaluation procedures, having been recruited from a consumer panel that frequently conducts sensory assessments, may have helped.

#### 4.1.2. Open Comments

The findings based on product feedback reveal clear and consistent differences in the type and richness of descriptions provided across the two evaluation settings. Across all four products, oral responses collected in the Sense-AV setting were notably longer, more expressive, and lexically more diverse than the written comments obtained in the sensory booth, which aligns with prior research across various fields [52,53,54,55,56]. These differences, discussed in more detail below, likely reflect cognitive and expressive factors associated with oral and written responses, as well as contextual factors related to the evaluation settings.

The word count of oral responses was, on average, twice that of written comments, with participants frequently using intensifiers, repetitions, and emotionally expressive language. These features are often interpreted as markers of stronger affective engagement and spontaneous language use. For example, expressions such as “really, really tasty” or “almost addictive” were more common in the Sense-AV condition and less so in the written format. This is consistent with psychological studies suggesting that speaking allows for more emotional and less filtered responses due to its immediacy and lower cognitive demand for structuring content compared to writing [56,57].

Oral responses also tended to include a greater number of contextual references and subjective impressions. Participants in the Sense-AV setting more frequently described consumption scenarios (e.g., “perfect for a summer barbecue”, “enjoyed in a pleasant bar environment”) or articulated how a product might pair with other foods (e.g., “goes well with the chips”). Such experiential framing is rarely captured in traditional sensory booths, where the focus tends to be more technical. This suggests that immersive food consumption evaluation may encourage consumers to engage more holistically with products, drawing upon past experiences and imagined future consumption. Interestingly, negative or critical remarks, such as comments about excessive saltiness and greasiness in the sausage or excessive sweetness and acidity in the orange nectar, were more frequently expressed in the oral condition. This may be due to the reduced social inhibition and increased spontaneity afforded by speech input, as well as a greater sense of presence and authenticity fostered by the Sense-AV immersive setting.

Additionally, the larger SDs observed in oral responses in comparison with written ones can be interpreted as a reflection of individual differences in verbal fluency, comfort with speaking aloud, and engagement with the Sense-AV system. These variations align with qualitative findings from the semi-structured interviews. While several participants described the voice-based input as natural and effortless, comparing it to sending a voice message, others reported discomfort and difficulty formulating coherent speech. These contrasting perspectives highlight the importance of considering individual preferences and communication styles in the design of sensory evaluation protocols.

### 4.2. Food Intake

To our knowledge, this is the first sensory study using XR technologies that measures food intake, which limits direct comparisons with existing literature. Nonetheless, our findings revealed a significant reduction in intake during the Sense-AV session compared to the sensory booth, which was notable for most products, including grilled sausage, chips, orange nectar and beer, but not for mayonnaise. This pattern encourages reflection on the possible mechanisms behind these differences.

The lower intake in the immersive session may stem from a combination of contextual and ergonomic factors. Despite the Sense-AV system successfully providing a more ecologically valid and engaging environment, it also introduced challenges that likely affected natural consumption behaviour. For instance, participants reported a significantly higher difficulty manipulating food items and interacting with the mobile device while wearing the HMD, as indicated by significantly lower manipulation scores in the MCRRQ compared to the booth session. Although the interaction was generally successful, the increased effort required may have discouraged *ad libitum* intake. Additionally, cognitive load imposed by manipulating the food items and performing tasks such as reading and responding on a mobile phone while wearing the HMD may have fragmented attention. This may have divided focus between the sensory evaluation and task management, likely reducing participants’ engagement with the food and further contributing to decreased intake.

The immersive sports bar environment itself may have negatively influenced *ad libitum* consumption through heightened social presence and self-awareness. Ambient sounds and realistic visual cues might have activated social norms related to restrained eating in public, leading participants to moderate their intake subconsciously. This psychological effect may have encouraged more controlled or self-conscious consumption, particularly among individuals sensitive to external observation.

The novelty of the Sense-AV system might also play a role. For many participants, this was their first experience with XR technologies, potentially shifting attention away from consumption towards exploration or adaptation, which could lead to reduced intake. Incorporating a familiarisation or dummy session might be beneficial in future studies to mitigate this effect [58]. However, in the present study, its omission was deliberate, as the objective was to validate the Sense-AV system with untrained participants and to assess their spontaneous reactions during initial exposure.

Notably, the food products that showed significant reductions in intake were those for which overall liking remained similar across environments. Conversely, mayonnaise, which exhibited increased liking in the Sense-AV session, maintained consistent intake. This pattern suggests that immersive environments may influence consumption amounts, especially when sensory engagement or ease of interaction is affected, despite an overall congruent product-context across all food products consumed.

### 4.3. Questionnaires

#### 4.3.1. Manipulation, Comprehension, Reading and Response

The results demonstrated that participants experienced significantly greater difficulty in all assessed aspects of the MCRRQ during the Sense-AV session compared to the sensory booth. Nevertheless, it is notable that positive response rates remained high for most factors, exceeding 90% for manipulation, understanding of information via audio, and providing verbal open comments. This indicates that, despite the challenges introduced by the immersive environment, participants were generally able to perform these essential sensory evaluation tasks effectively. In particular, the high success rate for manipulating products and utensils represents a significant improvement compared to VR systems that rely solely on 3D development, where interaction is often limited or unnatural due to the absence of real tactile feedback (Table S1). In the present system, participants not only had a realistic visualisation of the products but were also able to physically manipulate them with ease. This seamless integration of real product handling within an immersive environment constitutes a key advantage of the approach, reinforcing the system’s validity.

However, interaction with the mobile phone proved considerably more challenging. Positive response rates for reading through the phone dropped markedly to 57.84%, while response rates via mobile phone were also reduced to 75.49%. These findings suggest that several visual and ergonomic factors contributed to these difficulties. During the interviews, some participants reported struggling to find the correct distance between the HMD and the mobile screen to achieve a clear view. In some instances, screen brightness, glare, or insufficient contrast made reading more difficult. The interface design may also have played a role, with dark text on a white background potentially being less effective in this context than a reversed contrast scheme. Additionally, individual factors such as damaged or scratched screens may have further impaired visibility. Improvements such as larger font sizes, stronger text contrast and formatting, and better calibration of mobile screen brightness could enhance legibility and overall interaction in future studies.

#### 4.3.2. Engagement

The analysis of the EQ revealed that overall engagement with the evaluation tasks remained high in both sessions. Internal consistency was acceptable in the Sense-AV session and moderate in the booth, supporting the reliability of the measurements across sessions. However, a small but statistically significant reduction was observed in Active Involvement in the Sense-AV session compared to the sensory booth. These results align with the findings of Hannum et al. [59], who reported that only the Active Involvement subfactor received significantly lower ratings in immersive conditions compared to the conventional sensory booth during wine evaluation. This result suggests that participants may have been slightly less focused or cognitively immersed in the task while in the immersive environment. Despite the interactive nature of the sports bar environment, the impact on attentional engagement was limited, and the observed reduction may reflect the higher mental workload imposed by navigating a novel XR setup rather than disengagement with the product evaluation itself [60,61].

No significant differences were observed for Purposeful Intent or Affective Value between sessions. This stability suggests that participants perceived the task as personally relevant and experienced similar emotional value from the evaluation regardless of the environment. Moreover, given that this was the first exposure to XR technologies for some participants, the maintenance of Purposeful Intent and Affective Value scores may also indicate a successful adaptation to the system itself.

#### 4.3.3. System Usability Scale (SUS) and Virtual Reality System Usability Questionnaire (VRSUQ)

The overall system usability, as measured by the SUS, achieved a score of 81.67, which falls within the Grade A range according to Sauro and Lewis [62] curved grading scale interpretation. This indicates a high level of usability, suggesting that users found the Sense-AV system to be effective, efficient, and satisfactory in supporting sensory evaluation tasks. Although the VRSUQ subscales (“Efficiency” and “Satisfaction”) showed slightly lower internal consistency, their scores were comparable to the SUS results, indicating a similarly positive UX. Given that the VRSUQ is a relatively new instrument and less widely validated, these findings should be interpreted with caution. Yet, they still reinforce the usability profile suggested by the SUS.

These quantitative SUS and VRSUQ results correspond closely with the qualitative feedback gathered from participants’ interviews. Most users described the experience as immersive and engaging, highlighting the virtual sports bar environment as familiar and comfortable, which likely contributed to the positive usability ratings. Initial minor issues, such as brief disorientation or HMD discomfort, were generally overcome through rapid adaptation, indicating that the system’s design supports intuitive use and reduces novelty effects over time. Practical aspects, such as the integration of real products and visible hand interactions, were positively received, although some challenges with depth perception and spatial configuration were reported. These insights highlight areas for technical refinement, which could further elevate usability and user satisfaction.

#### 4.3.4. Presence and Sensory Awareness

The results from the PSAQ revealed generally high levels of perceived presence across all four subscales, indicating that participants experienced the Sense-AV environment as both immersive and sensorially engaging. Physical Presence obtained the highest mean score (5.56 ± 1.46), followed closely by the Sensory Awareness component (5.52 ± 1.40), suggesting that participants felt physically situated within the virtual sports bar and were strongly engaged with the multisensory aspects of the testing environment. The Sense-AV system provided coherent visual and auditory stimuli, such as ambient noise, television screens showing football and tennis matches, and scenes of human interaction with audible conversations which reinforced the participants’ sense of “being there”. In addition, the ability to smell the actual food products, especially those with a more intense smell and requiring preparation, such as sausage and chips, contributed to a heightened sense of immersion and sensory engagement. These results are supported by individual interview data, which revealed that participants often perceived the environment as realistic and coherent, attributing their sense of presence to elements such as ambient sounds, the bar’s décor, and the apparent social activity within the space. This was reflected in participant comments such as “*The environment was pleasant, it was almost as if I were sitting at one of those tables with people around me. I felt completely at ease*” (P21, male, age 60), and “*You could tell that people were talking to each other. I found it amusing when they threw bottles into the bin and you could hear the sound, as if the bottle were really being thrown away*” (P8, female, age 50). These findings are consistent with prior research highlighting the importance of audiovisual fidelity and sensory congruence in fostering presence, which significantly influences the overall UX [63,64]. Self-Presence (5.27 ± 1.48) also exhibited relatively high scores, indicating that participants retained a sense of bodily and behavioural continuity within the Sense-AV setting, perceiving themselves as active evaluators of real food products in a convincing context. This sense of continuity between the real and virtual worlds may have been supported by the hybrid nature of the system, which maintained physical interaction with real food products.

Social Presence received the lowest score among the PSAQ dimensions (5.06 ± 1.80), though this value still reflects a generally positive experience. The relatively lower score may be attributed to the absence of reciprocal interaction with the people depicted in the virtual footage. While these persons contributed to the realism and contextual atmosphere of the bar, some participants reported a sense of social detachment, as one noted, “It felt like I was there, and no one was aware of my presence” (P12). These perceptions suggest that, for certain individuals, visual and contextual cues alone may be insufficient to fully establish mutual social engagement. Such experiences reflect the importance of dynamic social interactions in XR technologies, as evidenced by previous consumer and sensory research on immersive environments [49,58,65].

#### 4.3.5. Effects of Age, Sex, and Experience on Questionnaire Responses

When examining the effects of age, sex, and prior VR or MR experience on questionnaire responses, no significant differences were observed for sex or experience across any of the measures, suggesting that the Sense-AV system was perceived as similarly usable and engaging regardless of these variables. However, notable age-related differences did emerge. Older participants (50–65 years) reported significantly higher scores than younger participants (18–49 years) on several subscales of the MCRRQ, particularly in aspects related to manipulation, reading, and responding within the Sense-AV session. They also rated Purposeful Intent higher in both sessions, but this difference was only statistically significant in the conventional booth session.

While these findings may seem counterintuitive given the common assumption that younger individuals are generally more comfortable with digital technologies, several factors may help explain this pattern. Older participants may have approached the task with greater patience or attentiveness, resulting in more favourable evaluations of system usability and interaction. Younger users, by contrast, may have been more sensitive to minor interface limitations, such as font clarity or ease of response on mobile devices, particularly within the immersive setting.

Motivational factors may have also contributed. The higher scores in Purposeful Intent among older participants may reflect a stronger sense of focus or perceived value in completing the task, which could have positively influenced their overall ratings. Notably, this age-related difference was already significantly present in the conventional booth session, where no immersive technology was involved, suggesting that the Sense-AV system itself may not be the primary driver of this difference. Instead, it is possible that older participants approached the study with a greater sense of purpose or intrinsic motivation, which may have shaped their experience and perception across both conditions.

Nonetheless, no significant differences were found between older and younger participants in the scores for the SUS or the VRSUQ subscales. This lack of age-related effects may be due to the broader and more general nature of these instruments, which may not have been sensitive enough to capture the technical and specific interactional nuances reflected in the MCRRQ.

### 4.4. Semi-Structured Individual Interviews

The use of the Sense-AV was positively received by most participants, who characterised the experience as engaging, immersive, and markedly different from conventional sensory booth test settings. This aligns with the literature indicating the potential of immersive technologies to enhance contextual relevance and emotional engagement in sensory analysis [31,46,66]. The virtual sports bar was perceived as familiar, which may have contributed to participant comfort and willingness to repeat the experience, possibly by facilitating a psychological state more consistent with natural food consumption contexts [27,58].

Initial impressions were generally favourable, although a small number of participants experienced brief disorientation or discomfort, particularly linked to the weight and ergonomics of the HMD. This highlights the importance of considering physical comfort in the design of XR experiences, especially for repeated or prolonged use [67]. Despite these challenges, rapid adaptation was regularly reported, suggesting that the interface design was intuitive and that novelty effects can diminish quickly.

Immersion and presence were core strengths of the system. Participants responded positively to the audiovisual fidelity and contextual coherence of the bar environment. The presence of other people in the virtual sports bar contributed to the sensation of being in a real social setting, which aligns with findings from previous XR studies [27,58,65]. However, the absence of reciprocal interaction with these people emerged as a notable limitation. Some participants reported feeling socially invisible, pointing to a gap between visual immersion and social engagement. Future iterations of the system may benefit from incorporating basic interactivity to enhance social presence and further enhance realism.

From a practical perspective, the integration of real products and the visibility of real hands within the virtual environment was largely successful and appreciated by the participants. However, some participants reported difficulties with depth perception, particularly at the outset, although these issues typically resolved as they interacted with the products. Additionally, a few participants suggested that the positioning and size of the table within the virtual space could be improved. The current placement, close to the bar’s entrance, was regarded as less optimal. Future iterations could consider adjusting the table’s position and exploring different shapes (e.g., circular) or materials (e.g., matching the dark wood and steel elements of the virtual environment) to enhance overall realism and congruence.

The suggestions offered by participants indicate a generally positive reception of the system, with most proposing only minor adjustments rather than fundamental changes. This suggests that the core design was well aligned with user expectations. Feedback predominantly focused on technical refinements, including improving visual clarity when using the mobile phone through the HMD, enhancing HMD comfort, and achieving better alignment and congruence of the physical table within the virtual environment. These aspects are critical for sustaining immersion, reducing physical load and ensuring user comfort. Despite the use of an 8 K resolution video camera in the recorded footage, some participants felt that the visual quality of the environment could be improved, particularly in terms of clarity in certain regions of the digital space. As such, filming in 12 K or 16 K resolution, or using a camera with a larger sensor size, could be considered. However, this would need to be weighed against the increased costs of video equipment and the larger file sizes that would result. While a few participants proposed alternative input methods, most considered the use of the mobile phone to be an effective and appropriate solution. Overall, the feedback from the participants reinforces the potential of the Sense-AV system as a viable and engaging tool for sensory evaluation, with particular value for enhancing ecological validity.

## 5. Limitations

This study is subject to a few important limitations. The use of participants’ mobile phones within the Sense-AV system was identified as the main issue in both the MCRRQ and the individual interviews. Adjustments such as increasing text size, applying bold formatting, or improving font legibility may enhance the UX. Additionally, the spatial congruence of the real table could be improved by using a round table with bar-typical dimensions, or by matching its surface materials and colours to those of the surrounding digital environment to enhance realism. An initial habituation session in the Sense-AV setting is also recommended, as this may facilitate better interaction and reduce the initial impact of the technology. Furthermore, participants in this study all completed the conventional sensory booth session before the Sense-AV system session, meaning that the order of exposure was not counterbalanced. Despite the implementation of a longer wash-out period up to four weeks, this may have introduced some order effects, such as increased familiarity with the products, learning or adaptation to the evaluation task, or greater comfort with the evaluation process in the second session. Future studies should consider balancing session order to minimise potential bias.

Looking ahead, future technological advancements should focus on improving HMD weight distribution and comfort. Reducing the material around the nose area could allow for the use of various types of glassware, such as standard, wine, sparkling, or whiskey glasses. This remains a limitation for sensory evaluations involving liquids, as the use of straws or narrow-necked bottles is currently necessary to avoid interference with the HMD, an issue common across existing hardware.

## 6. Conclusions

This study displayed and validated the Sense-AV system, an AV solution designed to enhance ecological validity in sensory and consumer science by integrating real food products into immersive food consumption environments. To our knowledge, Sense-AV is the first XR system in this scientific field to integrate high-quality 360° real-world videos, physical food interaction and consumption, and autonomous mobile-based sensory evaluations, achieving a level of technical and experiential integration that has not been previously attained.

Participants generally reported high levels of presence, immersion, and sensory awareness. The virtual sports bar environment was consistently described as realistic and emotionally engaging, evoking familiar social experiences. The high-quality visuals, natural audio, and contextual congruence contributed to a believable and immersive setting, as reflected in both questionnaire scores and individual interview responses. In addition, participants were able to complete the sensory evaluation tasks independently using their own mobile devices. While certain aspects, such as screen readability through the HMD, presented major initial challenges, most users adapted quickly. Overall, the system supported an intuitive and natural sensory evaluation assessment, confirmed by high positive response rates and consistent product interaction. Notably, the verbal feedback collected during the immersive session was substantially richer and more expressive than the written comments in the conventional sensory booth condition, featuring greater use of adjectives, intensifiers, and contextual references. Thus, immersive settings, when combined with verbal feedback, may foster more spontaneous and emotive consumer insights. Additionally, the system achieved a Grade A on the SUS, along with high ratings on both the Efficiency and Satisfaction subscales of the VRSUQ. These results confirm that the system was perceived as highly usable, efficient, and satisfying to interact with.

From a sensory perspective, overall liking scores for most of the products did not differ significantly between the Sense-AV session and the conventional sensory booth, except for mayonnaise, which received significantly higher ratings in the immersive setting. This could be explained by the lower baseline score for mayonnaise in the booth, making the contrast with the immersive context more noticeable. The casual, social sports bar environment, combined with the physical manipulation of mayonnaise from a top-down bottle, may have enhanced the realism of the experience, contributing to its increased appeal. In contrast, food intake was significantly lower in the immersive condition for most products, except for mayonnaise. This reduction in intake may be attributed to the novelty of the system, limited familiarity with eating while wearing an HMD, as well as social or environmental factors. Future studies may address this by incorporating dummy sessions to help participants become more familiar with the system, potentially reducing the novelty effect and promoting more natural consumption behaviour.

## 7. Patents

All authors are listed as inventors on a Portuguese Provisional Patent Application (*Sistema de integração de elementos reais e comunicação externa em ambientes virtuais imersivos; no. 120499*) submitted to the Portuguese Institute of Industrial Property (*Instituto Nacional da Propriedade Industrial—INPI*), concerning the system described in this manuscript.

## Figures and Tables

**Table 1:** Mean overall liking scores (±SD) of the food products evaluated during the sensory booth and Sense-AV sessions.

Products	Overall Liking		*p* Value *
	Laboratory (Booth)	Sense-AV System	
Sausage	8.22 (±0.74)	8.20 (±0.70)	0.869
Mayonnaise	7.78 (±1.19)	8.01 (±0.88)	**0.019**
Nectar	8.11 (±1.10)	8.10 (±0.96)	0.794
Beer	7.94 (±1.29)	8.00 (±0.89)	0.850

* according to the Wilcoxon test.

**Table 2:** Mean intake (± SD) of the food products consumed during the sensory booth and Sense-AV sessions.

Products	Intake		*p* Value *
	Laboratory (Booth)	Sense-AV System	
Sausage (initial serving: 41.22 ± 1.84 g)	31.50 (±9.94) g	24.69 (±11.85) g	**<0.001**
Mayonnaise (nominal capacity: 450 mL)	7.61 (±5.73) g	6.54 (±4.79) g	0.091
Chips (initial serving: 46.63 ± 14.37 g)	23.24 (±10.95) g	19.86 (±15.16) g	**0.023**
Nectar (initial serving: 200 mL)	151.02 (±49.97) mL	102.52 (±53.65) mL	**<0.001**
Beer (initial serving: 200 mL)	144.12 (±44.90) mL	109.92 (±47.04) mL	**<0.001**

* according to the Wilcoxon test.

**Table 3:** Mean scores (± SD) and relative frequency of positive responses (scores ≥ 5) for the MCRRQ subscales evaluated during the sensory booth and Sense-AV sessions.

MCRRQ Factor	Session		*p* Value *
	Laboratory (Booth)	Sense-AV System	
Manipulation	6.74 (±0.42)/98.88%	5.97 (±0.96)/90.20%	**<0.001**
Reading through the phone	6.62 (±0.63)/99.02%	4.70 (±1.87)/57.84%	**<0.001**
Response through the phone	6.68 (±0.61)/99.02%	5.38 (±1.71)/75.49%	**<0.001**
Understanding the info (Booth—provided by the assistant; Sense-AV—provided by voice prompts)	6.86 (±0.34)/100%	6.67 (±0.63)/98.04%	**0.002**
Providing the open comment (Booth—written; Sense-AV—verbal)	6.78 (±0.50)/100%	6.26 (±1.04)/91.18%	**<0.001**

* according to the Wilcoxon test.

**Table 4:** Mean scores (± SD) for the EQ factors evaluated during the sensory booth and Sense-AV sessions.

EQ Factor	Session		*p* Value *
	Laboratory (Booth)	Sense-AV System	
Active Involvement	19.57 (±3.11)	19.08 (±2.77)	**0.024**
Purposeful Intent	26.54 (±2.03)	26.22 (±2.35)	0.114
Affective Value	18.70 (±2.45)	18.84 (±2.41)	0.616

* according to the Wilcoxon test.

**Table 5:** Mean scores (± SD) for questionnaire measures by age group and session.

Questionnaires	Age		*p* Value *
	Younger Consumers (18–49) (*n* = 52)	Older Consumers (50–65) (*n* = 50)	
MCRRQ			
Manipulation (Booth)	6.70 (±0.43)	6.78 (±0.40)	**0.042**
Manipulation (Sense-AV)	5.67 (±1.09)	6.28 (±0.69)	**0.005**
Reading through the phone (Booth)	6.50 (±0.72)	6.73 (±0.49)	0.113
Reading through the phone (Sense-AV)	4.26 (±1.86)	5.16 (±1.80)	**0.011**
Response through the phone (Booth)	6.56 (±0.72)	6.79 (±0.45)	0.090
Response through the phone (Sense-AV)	4.98 (±1.77)	5.81 (±1.56)	**0.003**
Understanding the info provided by the assistant (Booth)	6.92 (±0.26)	6.79 (±0.40)	0.061
Understanding the info provided by voice prompts (Sense-AV)	6.60 (±0.68)	6.73 (±0.56)	0.325
Providing the written comment (Booth)	6.81 (±0.52)	6.75 (±0.48)	0.279
Providing the verbal comment (Sense-AV)	6.13 (±1.20)	6.40 (±0.81)	0.467
EQ			
EQ—Active Involvement (Booth)	19.41 (±3.03)	19.73 (±3.24)	0.079
EQ—Active Involvement (Sense-AV)	18.62 (±3.25)	19.57 (±2.08)	0.253
EQ—Purposeful Intent (Booth)	26.15 (±2.31)	26.95 (±1.6)	**0.030**
EQ—Purposeful Intent (Sense-AV)	25.86 (±2.76)	26.59 (±1.79)	0.253
EQ—Affective Value (Booth)	18.62 (±2.29)	18.77 (±2.65)	0.482
EQ—Affective Value (Sense-AV)	18.84 (±2.56)	18.83 (±2.28)	0.751
SUS score	80.75 (±15.65)	82.65 (±13.18)	0.796
VRSUQ			
VRSUQ Efficiency score	83.54 (±14.61)	85.26 (±14.54)	0.538
VRSUQ Satisfaction score	85.42 (±16.99)	89.22 (±14.62)	0.264
PSAQ			
PSAQ—Physical Presence	5.51 (±1.12)	5.61 (±1.27)	0.489
PSAQ—Social Presence	5.08 (±1.22)	5.02 (±1.29)	0.891
PSAQ—Self-Presence	5.12 (±1.21)	5.42 (±1.36)	0.136
PSAQ—Sensory Awareness	5.34 (±1.05)	5.70 (±0.95)	0.067

* according to the Mann–Whitney U test.

## Data Availability

The data presented in this study are available upon request from the corresponding author due to consumer data privacy concerns.

## Supplementary Materials

The following supporting information can be downloaded at: https://www.mdpi.com/article/10.3390/foods14223950/s1, Table S1. Overview of XR studies on sensory and consumer science; Table S2. Question on Familiarity with Virtual Reality (VR) and Mixed Reality (MR) (PT–EN); Table S3. Manipulation, Comprehension, Reading, and Response Questionnaire (MCRRQ) (PT–EN); Table S4. Engagement Questionnaire (adapted from Hannum and Simons [41]) (PT-EN); Table S5. Virtual Reality System Usability Questionnaire (VRSUQ) Efficiency and Satisfaction subfactors (based on Kim and Rhiu [44]) (PT-EN); Table S6. Presence and Sensory Awareness Questionnaire (PSAQ) (based on the Multimodal Presence Scale from Makransky et al. [45] with an additional factor on Sensory Awareness from Bangcuyo et al. [46]) (PT-EN); Table S7. Semi-structured individual interview questions (PT-EN); Table S8. Mean scores (± SD) for individual items of the System Usability Scale (SUS) (from Brooke [42]) in the Sense-AV session; Table S9. Mean scores (± SD) for individual items of the VRSUQ Efficiency and Satisfaction subfactors (Kim and Rhiu [44]) in the Sense-AV session; Table S10. Mean scores (± SD) for individual items of the PSAQ subfactor (based on the Multimodal Presence Scale from Makransky et al. [45] with an additional factor on Sensory Awareness from Bangcuyo et al. [46]); Video S1. Participant consuming the products while using the Sense-AV system.

## Appendix A

### Appendix A.1. Hardware Configuration

The Sense-AV system is built upon a high-performance hardware infrastructure designed to support immersive audiovisual presentation, high-fidelity, low-latency HMD camera pass-through, and multimodal data collection (e.g., sensory evaluation questionnaire responses, audio comments, POV video recordings, and eye-tracking data). The Varjo XR-4 focal edition HMD (Varjo Technologies Oy, Helsinki, Finland) constitutes the central component of the setup, featuring dual mini-LED displays with 3840 × 3744 pixels per eye resolution, a wide 120° × 105° field of view (FOV), dual 20 MP front-facing cameras for pass-through integration, and up to 90 Hz refresh rate for smooth visual feedback (Figure 1a). The HMD features 200 Hz eye-tracking, integrated speakers, noise-cancelling microphones, and an open API for developers, enabling integration with external systems, facilitating a flexible UX, and simplifying data transfer and analysis [68]. The Varjo XR-4 HMD is connected to a high-performance workstation (Figure 1b) featuring an NVIDIA RTX 4090 GPU (NVIDIA Corporation, Santa Clara, CA, USA), an Intel Core i9 14900F processor (Intel Corporation, Santa Clara, CA, USA), and 64 GB of RAM, ensuring minimal latency and consistent performance, while enabling rapid HMD pass-through and video display. For positional tracking, two SteamVR Base Stations 2.0 (HTC Corporation, Taipei, Taiwan) (Figure 1c) are strategically positioned at opposite top corners of the chroma key structure, angled to ensure full front and side coverage of the participant’s sensory testing area. These stations provide precise external tracking of the HMD, compensating for the limitations of the Varjo XR-4’s beta inside-out tracking, which required the use of external stations to ensure reliable spatial positioning during the initial test trials.

During the sensory tests, the participant’s mobile device with internet connectivity plays an important role in the setup by linking the custom-developed application, the HMD, and Sense Test’s internal data collection system (SenseGest). This device ensures seamless communication and data synchronisation, enabling participants to complete sensory evaluation questionnaires (e.g., hedonic, descriptive, emotion evaluations, CATA [Check-All-That-Apply], TCATA [Temporal Check-All-That-Apply], and RATA [Rate-All-That-Apply]) while immersed in the virtual environment.

### Appendix A.2. Software Development, Data Capture and Synchronisation

The Sense-AV system’s software architecture integrates proprietary and third-party tools to ensure the efficient and synchronised execution of the UX outlined above. The AV application was developed using the Unity Game Engine (version 2022.3.42) (Unity Technologies, San Francisco, CA, USA), which served as the platform for presenting the pre-recorded virtual food consumption scenarios within the interactive framework. The application incorporates a designated “real-world interaction zone”, spatially anchored via the Varjo Marker positioned on the testing table. This region guarantees stable and consistent visualisation of real-world elements, regardless of their colour, including blue elements. By explicitly excluding chroma key filtering within this zone, potential visual artefacts are avoided, ensuring that critical real elements such as participants’ hands, clothing, mobile devices, or blue-coloured food or beverage products always remain visible while in this zone. The dimensions of the interaction zone are configurable, allowing adaptation to different table sizes and experimental setups. Additionally, a configuration file was implemented to dynamically adjust the angular thresholds governing the transition from AV to virtual-only display. This ensures that when a participant looks beyond the blue chroma-mapped area, such as behind or above them, they continue to perceive a coherent extension of the virtual environment. Although physical interaction is not possible in these peripheral regions, the visual continuity helps maintain immersion and prevents perceptual breaks. Furthermore, the application allows for the configuration of the video’s viewpoint, enabling the selection of which portion of the 360° footage will be displayed as the front view for the participant.

The virtual scenarios were captured using the Insta360 X4 camera (Arashi Vision Inc., Shenzhen, China), which records at a native resolution of 7680 × 3840 pixels at 30 fps. In post-production, the footage was upscaled to 8192 × 4096 pixels to enhance visual fidelity. Audio was recorded simultaneously to ensure spatial and temporal synchrony with the visual stimuli. Filming specifications were defined in advance to optimise the sense of presence, including the adoption of a minimum clearance radius of 1.5 metres around the camera to avoid artefacts or occlusions. Recordings were conducted using a tripod positioned at the average seated eye level of an adult participant, ensuring that the first-person perspective accurately reflected the natural viewpoint of seated users, consistent with sensory evaluation practices.

The Varjo Base v. 4.10 software (Varjo Technologies Oy, Helsinki, Finland) manages the participant’s eye-tracking configuration and provides access to advanced technical settings, including chroma key calibration parameters and display features. Eye-tracking data and POV video recordings are also captured directly via Varjo Base. These data are saved locally on the workstation, in MP4 format for video and CSV format for gaze data, for subsequent analysis.

Questionnaires were administered via the SenseGest system, which is accessible on each participant’s mobile device after individual login. As previously described, the system was synchronised with the Unity application over a local network using a custom API architecture that integrates the HMD, the Unity application, and SenseGest. This configuration enabled time-stamped logging of participant responses, allowing precise correlation between subjective ratings (e.g., hedonic evaluations) and the visual or auditory stimuli presented during each tasting trial. Recording of voice open comments occurs through the HMD, triggered by the Unity application. As all data are time-stamped, each recording and questionnaire response can be unambiguously linked to the specific sample or product being evaluated. Questionnaire responses (entered via mobile devices) and the corresponding audio comments are stored in the internal SenseGest database and can be exported for subsequent analysis. Additionally, SenseGest includes text-to-speech technology, ensuring verbal communication through a customised voice delivered via the HMD, without interrupting the spatial audio of the virtual environment and thereby preserving immersion. This voice provided a range of prompts, including a welcome message, instructions on what to do, and a closing message at the end of the evaluation session.

### Appendix A.3. Environmental Setup

The sensory evaluation sessions are conducted within a modular three-walled blue chroma key structure (2.0 × 2.3 × 1.0 m; width × height × depth) (Figure 1d), comprising a flat frontal panel and two perpendicular lateral panels with right-angled corners.

A softbox-equipped LED light (Figure 1e) is strategically positioned to uniformly illuminate both the chroma key structure and the participant area (Figure 1f). It is carefully calibrated to ensure that physical elements (e.g., food, hands, utensils) are clearly visible through the XR-4 pass-through, while avoiding overexposure or glare. The light’s intensity and colour temperature are also adjustable to match the virtual environment presented to the participant during the sensory evaluation.

A standard table and chair are positioned in the centre of the chroma key structure. To enhance congruence with the virtual environment, a cloth or surface covering may be placed on top of the table. The Varjo Marker is positioned on the table (or on the cloth or surface covering, if used) to spatially anchor the ‘real-world interaction zone’ described earlier. During the evaluations, food and beverage products, along with any required utensils, are placed on the table by the technician, who actively monitors the product sequence using an auxiliary tablet device.
