# Supplementary material for: High-Quality, High-Impact Augmented Virtuality System for the Evaluation of the Influence of Context on Consumer Perception and Hedonics: A Case Study in a Sports Bar Environment
Source: Foods. 2025 Nov 18;14(22):3950. doi: 10.3390/foods14223950 (PMC12652980; doi:10.3390/foods14223950)
Supplement: Supplementary file 1 [file foods-14-03950-s001.zip › SenseVirtual_SupplementaryInformation_Foods_Rev.pdf]

Supplementary material to the manuscript:

High-Quality, High-Impact Augmented Virtuality System for the Evaluation of the Influence of Context on Consumer Perception and Hedonics: A Case Study in a Sports Bar Environment

João Pedro Marques [1,2,3], José Carlos Ribeiro [1], Rui Costa Lima [2], Luís Baião [1,2], Bruna Barbosa [1,2,3], Célia Rocha [1,2] and Luís Miguel Cunha [1,*]

[1] GreenUPorto—Sustainable Agrifood Production Research Centre/Inov4Agro, DGAOT, Faculty of Sciences, University of Porto, Campus de Vairão, Rua da Agrária 747, 4485-646 Vairão, Portugal; joribeiro@fc.up.pt (J.C.R.)
[2] Sense Test, Lda, Rua Zeferino Costa 341, 4400-345 Vila Nova de Gaia, Portugal; rcl@sensetest.pt
[3] Faculty of Nutrition and Food Sciences, University of Porto, Rua do Campo Alegre 823, 4150-180 Porto, Portugal
* Correspondence: lmcunha@fc.up.pt; Tel.: +351-252660400

Table S1 – Overview of XR studies on sensory and consumer science.

XR type	Study field	Technological system	Digital environment format	Digital environments	Food products in XR	Study	Year
**AR**	Sensory	HoloLens 2	3D development	1) Enhanced palm tree and coconut setting 2) Enhanced pasture and cow setting	Yoghurts	Dong, *et al.* [1]	2021
**AR**	Sensory	Varjo XR-3 (with additional olfactory and haptic augmentation equipment)	NA	NA	1) Meatball 2) Plant-based ball with visual, olfactory and haptic augmentations	Vanhatalo, *et al.* [2]	2024
**AV**	Sensory	Oculus Rift CV1 (paired with helmet-mounted RealSense SR300)	3D development	1) Kitchen 2) Public park	1) Cookies 2) Pastry 3) Lemon mousse 4) Energy drink	Korsgaard, *et al.* [3]	2019
**AV**	Sensory	Microsoft HoloLens	360° video	Café	Tea-break snacks	Low, *et al.* [4]	2021
**AV**	Sensory	Meta Quest Pro	3D development	1) Sensory booth 2) Non-textured restaurant 3) Full-textured restaurant	1) Pasta dish 2) Grapes 3) Cookies 4) Glass of water	Long, *et al.* [5]	2023
**AV**	Sensory	Lenovo Explorer (paired with 120° Full HD wide-angle webcam)	1) 360° videos 2) 3D development (table and questionnaires only)	1)Living room 2) Food court	Nectars	Ribeiro, *et al.* [6]	2024
**VR**	Consumer (food choice)	Oculus Rift DK 2 (paired with hand-tracking system)	3D development	Buffet	1) VR carrot 2) VR pasta 3) VR chicken	Ung, *et al.* [7]	2018
**VR**	Consumer (food choice)	HTC Vive	3D development	Food court	VR food menus	Allman-Farinelli, *et al.* [8]	2019
**VR**	Consumer (food choice)	Oculus Rift DK2	3D development	Grocery store	VR general food	Lombart, *et al.* [9]	2019
**VR**	Consumer (food choice)	Oculus Rift DK 2 (with HTC Vive controllers)	3D development	Supermarket	VR breakfast cereal packaging	Siegrist, *et al.* [10]	2019
**VR**	Consumer (food choice)	ND	3D development	Supermarket	VR general food	Schnack, *et al.* [11]	2019

XR type	Study field	Technological system	Digital environment format	Digital environments	Food products in XR	Study	Year
**VR**	Consumer (food choice)	HTC VIVE Pro (paired with controllers)	3D development	Empty room (with a grocery store display table)	VR breakfast cereal packaging	Xu, *et al.* [12]	2021
**VR**	Consumer (food choice)	Oculus Quest 1 and Quest 2	3D development	Living room	VR general food	Plechatá, *et al.* [13]	2022
**VR**	Consumer (food disgust)	HTC Vive	3D development	1) No context (control)<br>2) Dog defecating	1) VR chocolate<br>2) Chocolate	Ammann, *et al.* [14]	2020
**VR**	Sensory (thinking-based)	Samsung Gear VR (paired with Galaxy S7 smartphone)	360º video	Beach	NA (only assessed the desire to drink different beverages)	Andersen, *et al.* [15]	2019
**VR**	Sensory	Samsung Gear VR (paired with Galaxy S6 smartphone)	360° videos	1) Bar<br>2) Winery	1) Beer<br>2) Sparkling wine	Picket and Dando [16]	2019
**VR**	Sensory	Trust Urban VR (paired with Galaxy S6 smartphone)	1) 3D development<br>2) 360° video	Pub	Beer	Sinesio, *et al.* [17]	2019
**VR**	Sensory	Oculus Go VR	360º videos	1) Sightseeing tour in 5-star hotel (relaxing environment)<br>2) Electronic music festival (noisy environment)	Chocolates	Kong, *et al.* [18]	2020
**VR**	Sensory	Oculus Go VR	360° videos	(1) Restaurant with bright lights (bright environment)<br>2) Restaurant with weakly glowing candles (dark environment)	Wine	Torrico, *et al.* [19]	2020
**VR**	Sensory (odour-based)	Oculus Go VR	360º images	1) No context (control)<br>2) Farm<br>3) City	Ground coffee	Barbosa Escobar, *et al.* [20]	2021
**VR**	Sensory	Oculus Go VR	360º videos	1) Restaurant<br>2) Countryside<br>3) City	1) Beef steak<br>2) Chocolate	Crofton, *et al.* [21]	2021
**VR**	Sensory	DELL visor VR headset (paired with controllers)	360º images	1) Forest (positive environment)<br>2) Old room (negative environment)	Chocolate	Torrico, *et al.* [22]	2021

XR type	Study field	Technological system	Digital environment format	Digital environments	Food products in XR	Study	Year
**VR**	Sensory	HTC Vive	3D development	1) Empty room (control)<br>2) Restaurant	Pizza rolls	Oliver and Hollis [23]	2021
**VR**	Sensory (appearance-based)	Oculus Rift S	3D development	Sensory booth	VR cakes	Alba-Martínez, *et al.* [24]	2022
**VR**	Sensory	HTC Vive (paired with tracking devices)	1) 360º video<br>2) 3D development (table, chairs, food products and questionnaires only)	Bar	Beer	Yang, *et al.* [25]	2022
**VR**	Sensory	Samsung Gear VR (paired with Galaxy S7 smartphone and audio headset)	360° video	1) Restaurant<br>2) Cinema	Rye bread	Song, *et al.* [26]	2022
**VR**	Sensory	Oculus Go VR	360° videos	1) Beach (summer environment)<br>2) Snow (winter environment)	1) Watermelon<br>2) Chocolate truffle<br>3) Cracker	Schouteten, *et al.* [27]	2024
**VR**	Sensory	HTC Vive Pro Eye	3D development	Sensory booth	Lemonade	Zulkarnain, *et al.* [28]	2024
**VR**	Sensory	Oculus Quest 2	3D development	Sensory booth	1) Juice<br>2) Biscuits	Zulkarnain, *et al.* [29]	2024
**VR**	Sensory (appearance-based)	Oculus Rift S	3D development	1) Dining Room<br>2) Back seat of a car	VR cakes	Alba-Martínez, *et al.* [30]	2024
**VR**	Sensory (odour-based)	HTC VIVE Pro Eye	3D development	Sensory booth	1) Sensory sticks (lemon, strawberry, cinnamon, vanilla, and caramel)<br>2) VR bakery products (pretzel, bread, croissant, baguette, and donut)	Zulkarnain, *et al.* [31]	2024

Table S2 – Question on Familiarity with Virtual Reality (VR) and Mixed Reality (MR) (PT–EN).

	PT – Por favor, indique o seu nível de familiaridade e experiência com tecnologias de imersão como realidade virtual ou realidade mista?	EN – Please indicate your level of familiarity and experience with immersive technologies such as virtual reality or mixed reality.
**Scale**	***1.*** *Nunca ouvi falar nem tive experiências com este tipo de tecnologias.*	**1.** I have never heard of or had any experience with this type of technology.
	2. *Já ouvi falar, mas nunca tive experiências com este tipo de tecnologias.*	**2.** I have heard of it but never had any experience with this type of technology.
	3. *Já experimentei este tipo de tecnologias pelo menos uma vez.*	**3.** I have tried this type of technology at least once.
	4. *Já experimentei este tipo de tecnologias ocasionalmente.*	**4.** I have used this type of technology occasionally.
	5. *Experimento este tipo de tecnologias regularmente.*	**5.** I use this type of technology regularly.

Table S3 – Manipulation, Comprehension, Reading, and Response Questionnaire (MCRRQ) (PT–EN).

PT – Por favor, indique o quão fácil foi realizar cada uma das seguintes tarefas, escolhendo entre 1 = "Extremamente difícil" e 7 = "Extremamente fácil":	**EN – Please indicate how easy it was to carry out each of the following tasks, choosing from 1 = "Extremely difficult" to 7 = "Extremely easy":**
Manipular (abrir, segurar) a cerveja/néctar.	Handling (opening, holding) the beer/nectar.
Beber a cerveja/nectar.	Drinking the beer/nectar.
Manipular (abrir a embalagem, pegar, adicionar às batatas fritas) a maionese.	Handling the mayonnaise (opening the container, picking it up, adding it to the chips).
Manipular (segurar) as batatas fritas.	Handling (holding) the chips.
Comer as batatas fritas.	Eating the chips.
Usar os talheres.	Using the cutlery.
Comer a salsicha.	Eating the sausage.
Ler informação no dispositivo móvel.	Reading information on the mobile device.
Responder aos questionários no dispositivo móvel.	Answering the questionnaires on the mobile device.
Perceber a informação que me foi fornecida (Sense-AV session - "pelo áudio"; Lab session - "pelo(a) técnico(a)").	Understanding the information provided (Sense-AV session – "via audio"; Lab session – "by the technician").
Fazer o meu comentário sobre a amostra (Sense-AV session - "por voz"; Lab session - "por escrito").	Giving my comment on the sample (Sense-AV session – "by voice"; Lab session – "in writing").

Table S4 – Engagement Questionnaire (adapted from Hannum and Simons (2020) [32]) (PT-EN).

	PT – Indique, por favor, o seu grau de concordância com as seguintes afirmações, escolhendo entre 1 = "Discordo totalmente" e 7 = "Concordo totalmente":	**EN – Please indicate your level of agreement with the following statements, choosing from 1 = "Strongly disagree" to 7 = "Strongly agree":**
**Active involvement**	*Perdi o interesse na prova.**	I lost interest in the task. *
	Estava distraído(a).*	I was distracted. *
	*Senti-me a perder a concentração durante a prova.**	I felt myself zoning out during the task. *
**Purposeful Intent**	*Considerei a prova significativa.*	I found the task meaningful.
	Senti-me empenhado(a) a completar a prova.	I felt dedicated to finishing the task.
	Eu quis dedicar toda a minha atenção à prova.	I wanted to devote my full attention to the task.
	O meu contributo foi significativo para os resultados da prova.	My contribution was significant to the outcome of the task.
**Affective Value**	*Achei a prova cativante.*	I found the task captivating.
	Durante a prova, estava a divertir-me.	During the task, I was enjoying myself.
	Senti-me motivado(a) para fazer um esforço extra durante a prova.	I was motivated to expend extra effort during the task.

*Reverse-coded item.

Table S5 – Virtual Reality System Usability Questionnaire (VRSUQ) Efficiency and Satisfaction subfactors (based on Kim and Rhiu (2024) [33]) (PT-EN).

	PT – Indique, por favor, o seu grau de concordância com as seguintes afirmações, escolhendo entre 1 = "Discordo totalmente" e 7 = "Concordo totalmente":	**EN – Please indicate your level of agreement with the following statements, choosing from 1 = "Strongly disagree" to 7 = "Strongly agree":**
**Efficiency**	*Consegui perceber perfeitamente a informação apresentada no ambiente virtual.*	I could clearly understand the information presented within the virtual environment.
	Penso que este sistema é de fácil utilização, simples de aprender e concebido de forma a que a maioria das pessoas se adapte facilmente a ele.	I think this system is user-friendly, straightforward to learn, and designed in such a way that most people will find it easy to adapt to.
	Penso que é fácil corrigir os erros cometidos durante as experiências neste sistema.	I think it is easy to correct errors made during experiences in this system.
**Satisfaction**	*Gostei da experiência deste sistema.*	I enjoyed the experience in this system.
	*Senti tonturas, enjoos ou dores de cabeça durante a utilização deste sistema. **	I felt dizzy, motion sickness, or a headache while experiencing this system. *
	*Durante a utilização deste sistema, senti constrangimentos psicológicos como tensão, frustração e pressão de tempo. **	While experiencing virtual reality, I felt mental burdens such as tension, frustration, and time pressure. *

*Reverse-coded item.

Table S6 – Presence and Sensory Awareness Questionnaire (PSAQ) (based on the Multimodal Presence Scale from Makransky et al. (2017) [34] with an additional factor on Sensory Awareness from Bangcuyo et al. (2015) [35]) (PT-EN).

	PT – Por favor, indique o seu grau de concordância com cada uma das seguintes afirmações, escolhendo entre 1 = "Discordo totalmente" e 7 = "Concordo totalmente":	**EN – Please state your degree of agreement on each of the following sentences, by choosing from 1 = "Strongly disagree" to 7 = "Strongly agree":**
**Physical Presence**	*O ambiente virtual parecia-me real.*	The virtual environment seemed real to me.
	Tinha a sensação de estar a atuar no ambiente virtual, em vez de observar algo de fora.	I had a sense of acting in the virtual environment, rather than observing something from the outside.
	A minha experiência no ambiente virtual parecia consistente com as minhas experiências no mundo real.	My experience in the virtual environment seemed consistent with my experiences in the real world.
	Enquanto estava no ambiente virtual, eu tive a sensação de "estar lá".	While I was in the virtual environment, I had a sense of "being there".
	Estava completamente cativado pelo mundo virtual.	I was completely captivated by the virtual world.
**Social Presence**	*Senti que estava na presença de outras pessoas no ambiente virtual.*	I felt like I was in the presence of other people in the virtual environment.
	Senti que as pessoas no ambiente virtual estavam cientes da minha presença.	I felt that people in the virtual environment were aware of my presence.
	As pessoas no ambiente virtual pareciam ser sencientes (conscientes e vivas) para mim.	The people in the virtual environment appeared to be sentient (conscious and alive) to me.
	Durante a experiência, a interação com outras pessoas no ambiente virtual foi tão fluida que não percebi a presença da tecnologia a mediar a comunicação.	During the experience, the interaction with other people in the virtual environment was so seamless that I didn't notice the presence of technology mediating the communication.
	Tive a sensação de que estava a interagir com outras pessoas no ambiente virtual, em vez de com uma simulação virtual.	I had a sense that I was interacting with other people in the virtual environment rather than a virtual simulation.
**Self-Presence**	*Senti-me como se o meu corpo real se estendesse naturalmente para o ambiente, como se pertencesse lá.*	I felt like my real body naturally extended into the environment, as if it belonged there.

	PT – Por favor, indique o seu grau de concordância com cada uma das seguintes afirmações, escolhendo entre 1 = “Discordo totalmente” e 7 = “Concordo totalmente”:	**EN – Please state your degree of agreement on each of the following sentences, by choosing from 1 = “Strongly disagree” to 7 = “Strongly agree”:**
	Quando algo acontecia ao meu corpo no ambiente virtual, sentia como se estivesse a acontecer ao meu corpo real.	When something happened to my body in the virtual environment, it felt like it was happening to my real body.
	Senti como se o meu braço real estivesse harmoniosamente integrado no ambiente, como se não houvesse qualquer barreira entre o meu corpo e o espaço à minha volta.	I felt like my real arm was seamlessly integrated into the environment, as if there were no barrier between my body and the space around me.
	Senti como se a minha mão real fosse naturalmente parte do ambiente, totalmente presente e capaz de interagir dentro dele.	I felt like my real hand was naturally part of the environment, fully present and able to interact within it.
	Durante a experiência, senti como se o meu corpo real e o ambiente se tornassem um só, como se estivesse fisicamente presente naquele espaço.	During the experience, I felt like my real body and the environment became one, as if I were physically present in that space.
**Sensory Awareness**	*Todos os meus sentidos estavam completamente envolvidos pelo ambiente de teste.*	All my senses were completely engaged by the testing environment.
	Os aspetos visuais do ambiente de teste envolveram-me completamente.	The visual aspects of the testing environment completely involved me.
	Os aspetos auditivos do ambiente de teste envolveram-me completamente.	The auditory aspects of the testing environment completely involved me.
	Os aspetos olfativos do ambiente de teste envolveram-me completamente.	The olfactory aspects of the testing environment completely involved me.

Table S7 – Semi-structured individual interview questions (PT-EN).

	PT	EN
**Initial Impressions**	*Por favor, descreva como se sentiu ao realizar a provas neste sistema de realidade mista? Achou cativante/divertido? Achou muito confuso? Gostaria de realizar mais provas neste tipo de sistema?*	Please describe how you felt while carrying out the test using this mixed reality system. Did you find it engaging or enjoyable? Did it seem confusing? Would you be interested in carrying out more tests using this type of system?
**Immersion and Presence**	*Achou o ambiente adequado para a realização da prova? Consegue descrever o ambiente?*	Did you find the environment appropriate for performing the test? Can you describe the environment?
	O quanto real lhe parecia o ambiente onde estava inserido? Sentia-se imerso e presente no ambiente?	How real did the environment feel to you? Did you feel immersed and present in it?
	O que achou da presença das outras pessoas no ambiente? Sentiu que aumentou o seu nível de imersão? Sentiu que elas estavam conscientes da sua presença?	What did you think about the presence of other people in the environment? Did it enhance your sense of immersion? Did you feel that they were aware of your presence?
	Sentiu que as suas mãos/braços e a mesa onde realizou a prova se enquadravam bem no ambiente virtual?	Did your hands/arms and the table used for the test feel well integrated into the virtual environment?
	Houve algum elemento do ambiente (visual, auditivo, social) que tenha sentido que não o envolveu ou que tenha levado a alguma perda de imersão/realismo?	Was there any element of the environment (visual, auditory, or social) that you felt did not engage you or led to a loss of immersion or realism?
**User Experience (UX)**	*Teve dificuldades e constrangimentos na manipulação e consumo dos diferentes produtos? Se sim, em quais produtos teve mais dificuldade?*	Did you experience any difficulties or discomfort when handling and consuming the different products? If so, with which products did you encounter the most difficulty?
	Teve dificuldades em responder aos questionários no telemóvel? Achou que a utilização do telemóvel nesta prova foi congruente com o que faria numa situação real? E relativamente a efetuar os comentários por áudio? Sentiu alguma dificuldade?	Did you experience any difficulty in responding to the questionnaires on your mobile phone? Did the use of the mobile phone during the test feel consistent with what you would do in a real-life situation? What about providing audio comments? Did you find that difficult?

	PT	EN
	Sentiu alguma dificuldade em acompanhar as instruções da prova através dos áudios?	Did you have any difficulty following the test instructions delivered through audio?
	Sentiu algumas dificuldades físicas ou mentais com a utilização do sistema de realidade mista?	Did you experience any physical or mental discomfort while using the mixed reality system?
**Recommendations**	*Tem algum comentário ou recomendação a fazer relativamente ao sistema de realidade mista?*	Do you have any comments or recommendations regarding the mixed reality system?

Table S8 – Mean scores (± SD) for individual items of the System Usability Scale (SUS) (from Brooke (1996) [36]) in the Sense-AV session.

SUS items	Mean scores (± SD)
I think that I would like to use this system frequently.	5.75 (± 1.46)
I found the system unnecessarily complex.*	2.11 (± 1.51)
I thought the system was easy to use.	6.21 (± 1.11)
I think that I would need the support of a technical person to be able to use this system.*	2.95 (± 1.90)
I found the various functions in this system were well integrated.	6.04 (± 1.15)
I thought there was too much inconsistency in this system.*	2.28 (± 1.53)
I would imagine that most people would learn to use this system very quickly.	5.61 (± 1.40)
I found the system very awkward to use.*	1.69 (± 1.21)
I felt very confident using the system.	6.08 (± 1.19)
I needed to learn a lot of things before I could get going with this system.*	1.66 (± 1.16)

*Reverse-coded item.

Table S9 – Mean scores (± SD) for individual items of the VRSUQ Efficiency and Satisfaction subfactors (Kim and Rhiu (2024)) in the Sense-AV session.

	VRSUQ subscale items	Mean scores (± SD)
Efficiency	I could clearly understand the information presented within the virtual environment.	6.40 (± 1.03)
	I think this system is user-friendly, straightforward to learn, and designed in such a way that most people will find it easy to adapt to.	5.94 (± 1.29)
	I think it is easy to correct errors made during experiences in this system.	5.84 (± 1.20)
Satisfaction	I enjoyed the experience in this system.	6.41 (± 0.95)
	I felt dizzy, had motion sickness, or a headache while experiencing this system. *	1.98 (± 1.57)
	While experiencing virtual reality, I felt mental burdens such as tension, frustration, and time pressure. *	1.73 (± 1.33)

*Reverse-coded item.

Table S10 – Mean scores (± SD) for individual items of the PSAQ subfactor (based on the Multimodal Presence Scale from Makransky et al. (2017) [34] with an additional factor on Sensory Awareness from Bangcuyo et al. (2015) [35]).

	PSAQ subfactor items	Mean scores (± SD)
Physical Presence	The virtual environment seemed real to me.	5.82 (± 1.32)
	I had a sense of acting in the virtual environment, rather than observing something from the outside.	5.03 (± 1.69)
	My experience in the virtual environment seemed consistent with my experiences in the real world.	5.50 (± 1.38)
	While I was in the virtual environment, I had a sense of "being there".	5.71 (± 1.35)
	I was completely captivated by the virtual world.	5.76 (± 1.37)
Social Presence	I felt like I was in the presence of other people in the virtual environment.	5.63 (± 1.36)
	I felt that people in the virtual environment were aware of my presence.	4.19 (± 2.11)
	The people in the virtual environment appeared to be sentient (conscious and alive) to me.	6.04 (± 1.19)
	During the experience, the interaction with other people in the virtual environment was so seamless that I didn't notice the presence of technology mediating the communication.	4.53 (± 1.78)
	I had a sense that I was interacting with other people in the virtual environment rather than a virtual simulation.	4.90 (± 1.71)
Self-Presence	I felt like my real body naturally extended into the environment, as if it belonged there.	5.30 (± 1.52)
	When something happened to my body in the virtual environment, it felt like it was happening to my real body.	5.01 (± 1.59)
	I felt like my real arm was seamlessly integrated into the environment, as if there were no barrier between my body and the space around me.	5.31 (± 1.38)
	I felt like my real hand was naturally part of the environment, fully present and able to interact within it.	5.40 (± 1.35)
	During the experience, I felt like my real body and the environment became one, as if I were physically present in that space.	5.30 (± 1.50)
Sensory Awareness	All my senses were completely engaged by the testing environment.	5.74 (± 1.27)
	The visual aspects of the testing environment completely involved me.	5.77 (± 1.11)
	The auditory aspects of the testing environment completely involved me.	5.96 (± 1.06)

PSAQ subfactor items	Mean scores (± SD)
The olfactory aspects of the testing environment completely involved me.	4.61 (± 1.70)

References

1. Dong, Y.; Sharma, C.; Mehta, A.; Torrico, D.D. Application of Augmented Reality in the Sensory Evaluation of Yogurts. *Fermentation* **2021**, *7*, 147, doi:10.3390/fermentation7030147.
2. Vanhatalo, S.; Lappi, J.; Rantala, J.; Farooq, A.; Sand, A.; Raisamo, R.; Sozer, N. Meat- and plant-based products induced similar satiation which was not affected by multimodal augmentation. *Appetite* **2024**, *194*, 107171, doi:10.1016/j.appet.2023.107171.
3. Korsgaard, D.; Bjøner, T.; Nilsson, N.C. Where would you like to eat? A formative evaluation of mixed-reality solitary meals in virtual environments for older adults with mobility impairments who live alone. *Food Research International* **2019**, *117*, 30-39, doi:10.1016/j.foodres.2018.02.051.
4. Low, J.Y.Q.; Lin, V.H.F.; Jun Yeon, L.; Hort, J. Considering the application of a mixed reality context and consumer segmentation when evaluating emotional response to tea break snacks. *Food Quality and Preference* **2021**, *88*, 104113, doi:10.1016/j.foodqual.2020.104113.
5. Long, J.W.; Masters, B.; Sajjadi, P.; Simons, C.; Masterson, T.D. The development of an immersive mixed-reality application to improve the ecological validity of eating and sensory behavior research. *Frontiers in Nutrition* **2023**, *10*, 1170311, doi:10.3389/fnut.2023.1170311.
6. Ribeiro, J.C.; Rocha, C.; Barbosa, B.; Lima, R.C.; Cunha, L.M. Sensory Analysis Performed within Augmented Virtuality System: Impact on Hedonic Scores, Engagement, and Presence Level. *Foods* **2024**, *13*, doi:10.3390/foods13152456.
7. Ung, C.-Y.; Menozzi, M.; Hartmann, C.; Siegrist, M. Innovations in consumer research: The virtual food buffet. *Food Quality and Preference* **2018**, *63*, 12-17, doi:10.1016/j.foodqual.2017.07.007.
8. Allman-Farinelli, M.; Ijaz, K.; Tran, H.; Pallotta, H.; Ramos, S.; Liu, J.; Wellard-Cole, L.; Calvo, R.A. A Virtual Reality Food Court to Study Meal Choices in Youth: Design and Assessment of Usability. *JMIR Formative Research* **2019**, *3*, e12456, doi:10.2196/12456.
9. Lombart, C.; Millan, E.; Normand, J.-M.; Verhulst, A.; Labbé-Pinlon, B.; Moreau, G. Consumer perceptions and purchase behavior toward imperfect fruits and vegetables in an immersive virtual reality grocery store. *Journal of Retailing and Consumer Services* **2019**, *48*, 28-40, doi:10.1016/j.jretconser.2019.01.010.
10. Siegrist, M.; Ung, C.-Y.; Zank, M.; Marinello, M.; Kunz, A.; Hartmann, C.; Menozzi, M. Consumers' food selection behaviors in three-dimensional (3D) virtual reality. *Food Research International* **2019**, *117*, 50-59, doi:10.1016/j.foodres.2018.02.033.
11. Schnack, A.; Wright, M.J.; Holdershaw, J.L. Immersive virtual reality technology in a three-dimensional virtual simulated store: Investigating telepresence and usability. *Food Research International* **2019**, *117*, 40-49, doi:10.1016/j.foodres.2018.01.028.
12. Xu, C.; Demir-Kaymaz, Y.; Hartmann, C.; Menozzi, M.; Siegrist, M. The comparability of consumers' behavior in virtual reality and real life: A validation study of virtual reality based on a ranking task. *Food Quality and Preference* **2021**, *87*, 104071, doi:10.1016/j.foodqual.2020.104071.
13. Plechatá, A.; Morton, T.; Perez-Cueto, F.J.A.; Makransky, G. A randomized trial testing the effectiveness of virtual reality as a tool for pro-environmental dietary change. *Sci Rep* **2022**, *12*, 14315, doi:10.1038/s41598-022-18241-5.
14. Ammann, J.; Hartmann, C.; Peterhans, V.; Ropelato, S.; Siegrist, M. The relationship between disgust sensitivity and behaviour: A virtual reality study on food disgust. *Food Quality and Preference* **2020**, *80*, 103833, doi:10.1016/j.foodqual.2019.103833.
15. Andersen, I.N.S.K.; Kraus, A.A.; Ritz, C.; Bredie, W.L.P. Desires for beverages and liking of skin care product odors in imaginative and immersive virtual reality beach contexts. *Food Research International* **2019**, *117*, 10-18, doi:10.1016/j.foodres.2018.01.027.
16. Picket, B.; Dando, R. Environmental Immersion's Influence on Hedonics, Perceived Appropriateness, and Willingness to Pay in Alcoholic Beverages. *Foods* **2019**, *8*, doi:10.3390/foods8020042.
17. Sinesio, F.; Moneta, E.; Porcherot, C.; Abbà, S.; Dreyfuss, L.; Guillamet, K.; Bruyninckx, S.; Laporte, C.; Henneberg, S.; McEwan, J.A. Do immersive techniques help to capture consumer reality? *Food Quality and Preference* **2019**, *77*, 123-134, doi:10.1016/j.foodqual.2019.05.004.
18. Kong, Y.; Sharma, C.; Kanala, M.; Thakur, M.; Li, L.; Xu, D.; Harrison, R.; Torrico, D.D. Virtual Reality and Immersive Environments on Sensory Perception of Chocolate Products: A Preliminary Study. *Foods* **2020**, *9*, 515, doi:10.3390/foods9040515.

19. Torrico, D.D.; Han, Y.; Sharma, C.; Fuentes, S.; Gonzalez Viejo, C.; Dunshea, F.R. Effects of Context and Virtual Reality Environments on the Wine Tasting Experience, Acceptability, and Emotional Responses of Consumers. *Foods* **2020**, *9*, 191, doi:10.3390/foods9020191.
20. Barbosa Escobar, F.; Petit, O.; Velasco, C. Virtual Terroir and the Premium Coffee Experience. *Frontiers in Psychology* **2021**, *12*, 586983, doi:10.3389/fpsyg.2021.586983.
21. Crofton, E.; Murray, N.; Botinestean, C. Exploring the Effects of Immersive Virtual Reality Environments on Sensory Perception of Beef Steaks and Chocolate. *Foods* **2021**, *10*, 1154, doi:10.3390/foods10061154.
22. Torrico, D.D.; Sharma, C.; Dong, W.; Fuentes, S.; Gonzalez Viejo, C.; Dunshea, F.R. Virtual reality environments on the sensory acceptability and emotional responses of no- and full-sugar chocolate. *LWT* **2021**, *137*, 110383, doi:10.1016/j.lwt.2020.110383.
23. Oliver, J.H.; Hollis, J.H. Virtual Reality as a Tool to Study the Influence of the Eating Environment on Eating Behavior: A Feasibility Study. *Foods* **2021**, *10*, doi:10.3390/foods10010089.
24. Alba-Martínez, J.; Sousa, P.M.; Alcañiz, M.; Cunha, L.M.; Martínez-Monzó, J.; García-Segovia, P. Impact of context in visual evaluation of design pastry: Comparison of real and virtual. *Food Quality and Preference* **2022**, *97*, 104472, doi:10.1016/j.foodqual.2021.104472.
25. Yang, Q.; Nijman, M.; Flintham, M.; Tennent, P.; Hidrio, C.; Ford, R. Improving simulated consumption context with virtual Reality: A focus on participant experience. *Food Quality and Preference* **2022**, *98*, 104531, doi:10.1016/j.foodqual.2022.104531.
26. Song, X.; Pérez-Cueto, F.J.A.; Bredie, W.L.P. Food Desires and Hedonic Discrimination in Virtual Reality Varying in Product–Context Appropriateness among Older Consumers. *Foods* **2022**, *11*, 3228, doi:10.3390/foods11203228.
27. Schouteten, J.J.; Van Severen, A.; Dull, D.; De Steur, H.; Danner, L. Congruency of an eating environment influences product liking: A virtual reality study. *Food Quality and Preference* **2024**, *113*, 105066, doi:10.1016/j.foodqual.2023.105066.
28. Zulkarnain, A.H.B.; Cao, X.; Kókai, Z.; Gere, A. Self-Assessed Experience of Emotional Involvement in Sensory Analysis Performed in Virtual Reality. *Foods* **2024**, *13*, 375, doi:10.3390/foods13030375.
29. Zulkarnain, A.H.B.; Kókai, Z.; Gere, A. Immersive sensory evaluation: Practical use of virtual reality sensory booth. *MethodsX* **2024**, *12*, 102631, doi:10.1016/j.mex.2024.102631.
30. Alba-Martínez, J.; Alcañiz, M.; Martínez-Monzó, J.; Cunha, L.M.; García-Segovia, P. Beyond Reality: Exploring the effect of different virtual reality environments on visual assessment of cakes. *Food Research International* **2024**, *179*, 114019, doi:10.1016/j.foodres.2024.114019.
31. Zulkarnain, A.H.B.; Kókai, Z.; Gere, A. Assessment of a virtual sensory laboratory for consumer sensory evaluations. *Heliyon* **2024**, *10*, e25498, doi:10.1016/j.heliyon.2024.e25498.
32. Hannum, M. E., & Simons, C. T. (2020). Development of the engagement questionnaire (EQ): A tool to measure panelist engagement during sensory and consumer evaluations. Food Quality and Preference, 81, 103840. https://doi.org/10.1016/j.foodqual.2019.103840
33. Kim, Y. M., & Rhiu, I. (2024). Development of a virtual reality system usability questionnaire (VRSUQ). *Applied Ergonomics*, 119, 104319. https://doi.org/10.1016/j.apergo.2024.104319
34. Makransky, G., Lilleholt, L., & Aaby, A. (2017). Development and validation of the Multimodal Presence Scale for virtual reality environments: A confirmatory factor analysis and item response theory approach. *Computers in Human Behavior*, 72, 276-285. https://doi.org/10.1016/j.chb.2017.02.066
35. Bangcuyo, R. G., Smith, K. J., Zumach, J. L., Pierce, A. M., Guttman, G. A., & Simons, C. T. (2015). The use of immersive technologies to improve consumer testing: The role of ecological validity, context and engagement in evaluating coffee. F*ood Quality and Preference*, 41, 84-95. https://doi.org/10.1016/j.foodqual.2014.11.017
36. Brooke, J. SUS - a "quick and dirty" usability scale. In *Usability Evaluation in Industry*; Taylor & Francis: London, UK, 1996; pp. 189-194.
